# Mesoscopic Kinetic Approach of Nonequilibrium Effects for Shock Waves

**DOI:** 10.3390/e26030200

**Published:** 2024-02-26

**Authors:** Ruofan Qiu, Xinyuan Yang, Yue Bao, Yancheng You, Hua Jin

**Affiliations:** School of Aerospace Engineering, Xiamen University, Xiamen 361005, China; yangxy@stu.xmu.edu.cn (X.Y.); bao_yue@foxmail.com (Y.B.);

**Keywords:** shock wave, nonequilibrium, Boltzmann equation, CFD, molecular motion

## Abstract

A shock wave is a flow phenomenon that needs to be considered in the development of high-speed aircraft and engines. The traditional computational fluid dynamics (CFD) method describes it from the perspective of macroscopic variables, such as the Mach number, pressure, density, and temperature. The thickness of the shock wave is close to the level of the molecular free path, and molecular motion has a strong influence on the shock wave. According to the analysis of the Chapman-Enskog approach, the nonequilibrium effect is the source term that causes the fluid system to deviate from the equilibrium state. The nonequilibrium effect can be used to obtain a description of the physical characteristics of shock waves that are different from the macroscopic variables. The basic idea of the nonequilibrium effect approach is to obtain the nonequilibrium moment of the molecular velocity distribution function by solving the Boltzmann–Bhatnagar–Gross–Krook (Boltzmann BGK) equations or multiple relaxation times Boltzmann (MRT-Boltzmann) equations and to explore the nonequilibrium effect near the shock wave from the molecular motion level. This article introduces the theory and understanding of the nonequilibrium effect approach and reviews the research progress of nonequilibrium behavior in shock-related flow phenomena. The role of nonequilibrium moments played on the macroscopic governing equations of fluids is discussed, the physical meaning of nonequilibrium moments is given from the perspective of molecular motion, and the relationship between nonequilibrium moments and equilibrium moments is analyzed. Studies on the nonequilibrium effects of shock problems, such as the Riemann problem, shock reflection, shock wave/boundary layer interaction, and detonation wave, are introduced. It reveals the nonequilibrium behavior of the shock wave from the mesoscopic level, which is different from the traditional macro perspective and shows the application potential of the mesoscopic kinetic approach of the nonequilibrium effect in the shock problem.

## 1. Introduction

In the realm of supersonic flow, shock waves stand out as one of the most typical phenomena, exerting a significant influence on the distribution of the flow field. In the development of supersonic aircraft and engines, the structure and properties of shock waves often take precedence. Pham-Van-Diep et al. [1], through experimental means, observed nonequilibrium molecular motion within shock waves. Such nonequilibrium molecular motion can induce nonequilibrium effects on different physical levels. The thickness of shock waves is often on the scale of the mean free path of molecules, and molecular motion plays a substantial role in influencing shock waves. Traditional approaches to understanding and studying shock waves mainly involve macroscopic variables such as the Mach number, pressure, density, and temperature, with little attention paid to nonequilibrium effects.

Macroscopic fluid control equations are the primary computational fluid dynamics (CFD) methods for studying supersonic flow, such as the Navier-Stokes and Euler equations. The latter is commonly considered a simplified equation obtained by neglecting viscous effects in the former. However, starting from the mesoscopic Boltzmann equation can lead to a different perspective. By performing Chapman-Enskog analysis on the Boltzmann–Bhatnagar–Gross–Krook (Boltzmann BGK) equations [2] or multiple relaxation times Boltzmann (MRT-Boltzmann) equations [3], macroscopic equations can be derived. If the nonequilibrium terms of the molecular velocity distribution function are neglected, retaining only the equilibrium terms, the Euler equation is obtained. Including the first-order nonequilibrium terms of the molecular velocity distribution function yields the Navier-Stokes equation. From this viewpoint, the Euler equation can be regarded as a simplified equation that neglects the nonequilibrium effects present in the Navier-Stokes equation. In other words, the additional terms in the Navier-Stokes equation compared to the Euler equation reflect the first-order nonequilibrium effects in the fluid system. Based on this perspective, focusing on the distribution characteristics of nonequilibrium effects in fluid systems allows for the exploration of phenomena and mechanisms that deviate the fluid system from equilibrium.

To effectively capture the nonequilibrium effects in fluid systems, the focus can be directed towards the Boltzmann-BGK equation and MRT-Boltzmann equation, as discussed earlier. These equations are methods based on nonequilibrium statistical mechanics that describe molecular motion statistics, providing a means to depict macroscopic fluid flow in multiscale physical systems. The fundamental idea involves the statistical migration and collisions of molecules to achieve the macroscopic fluid flow description, with collision processes achieved through the difference between the current state of the molecular velocity distribution function and its corresponding local equilibrium state, representing the nonequilibrium effects of molecular velocity distribution. Clearly, nonequilibrium characteristics, such as those in the Boltzmann-BGK equation and MRT-Boltzmann equation, are well-suited for studying nonequilibrium effects in fluid systems.

The Lattice Boltzmann Method (LBM) [4,5,6] is a mesoscopic CFD method for solving the Boltzmann-BGK equation or MRT-Boltzmann equation. Due to its clear physical interpretation, straightforward boundary handling, and efficient parallel computing capabilities, LBM has found widespread applications in incompressible flow problems such as turbulence [6,7], multiphase flow [8,9], heat transfer [10,11], fluid-structure interaction [12,13], and porous media flow [14,15]. However, standard LBM models are limited by the construction of the discrete velocity model and are not suitable for compressible flow with shock waves. Over the years, significant developments have been made in discrete velocity models, equilibrium distribution functions, and solution formats to achieve compressible LBM. Various compressible LBM models have been established based on different approaches, enabling numerical simulations of supersonic flows with shock waves [16,17,18,19,20,21,22,23]. However, most research on compressible LBM has primarily focused on establishing theoretical frameworks and models to achieve the required physical accuracy. When LBM is merely used as a solving method for the Navier-Stokes equations, it does not delve into the statistical molecular motion information. The study of nonequilibrium effects utilizes the mesoscopic nature of LBM. In recent years, Professor Xu’s research group has conducted extensive work in nonequilibrium theory. They have analyzed the relationship between nonequilibrium moments and macroscopic quantities such as viscous stress and heat flux. Additionally, their research has covered nonequilibrium behaviors in phenomena such as multiphase flow [8,24,25,26], fluid instability [25,27], shock waves [17,28], and chemical reactions [3,29,30,31].

Through the LBM, various nonequilibrium moments can be obtained, each reflecting nonequilibrium effects on the corresponding physical level. These nonequilibrium effects serve as indicators of the deviation of the fluid system from equilibrium, providing valuable information. In addition to LBM, mesoscopic methods [32,33] such as Direct Simulation Monte Carlo (DSMC) [34] and the discrete velocity method (DVM) [35] can also be used for nonequilibrium effect studies. DSMC is a stochastic method, and it is well suited for simulations of high-speed rarefied gas flows. Significant statistical noise and slow convergence speeds may arise when simulating low-speed flows, attributed to the statistical nature of the method [36]. Due to the separate handling of streaming and collision processes in DSMC, simulating near-continuum flows becomes computationally expensive as the spatial cell size and time step must be smaller than the mean free path and mean collision time of gas molecules for numerical accuracy to be guaranteed [37]. DVM is a deterministic method that can simulate fluid flows from the free molecular regime to the continuum regime by directly dividing discrete velocity points in velocity space. However, a large number of discrete velocity points are usually required to minimize quadrature error, making it computationally more intensive compared to LBM, especially for fluid flows near the continuum regime. Unlike DVM, LBM significantly reduces computational costs by seeking minimal velocities and discrete distribution functions in particle velocity space instead of using the full Maxwellian distribution [38]. The desired physical conservation laws can be exactly satisfied through weighted sums of distribution functions at these velocity points. In LBM, this set of discrete velocities and corresponding equilibrium distribution functions is referred to as the lattice Boltzmann model. Currently, most studies on shock waves using DSMC and DVM focus on macroscopic quantities such as density, velocity, and temperature; few instances in the literature have discussed the physical significance of shock waves from nonequilibrium effects caused by molecular motion deviating from the equilibrium state. Based on existing literature, numerical investigations specifically addressing this perspective primarily employ LBM. Thus, this paper primarily introduces the research methodology based on LBM for studying these nonequilibrium effects of shock waves. Specifically, it focuses on a nonequilibrium theory based on nonequilibrium moments, presenting an equation form and an approach to understanding nonequilibrium effects from the perspective of molecular motion. The emphasis is placed on the numerical values and the physical implications of the nonequilibrium moments. Through this methodology, a novel understanding of shock wave phenomena can be gained by examining the factors that drive the fluid system away from equilibrium.

The structure of this paper is as follows: In the first section, the relationship between LBM nonequilibrium moments and macroscopic equations is outlined. The second section introduces an approach to understanding nonequilibrium effects from the perspective of molecular motion. The third section demonstrates the application of the nonequilibrium effects method in studying shock wave problems. Finally, the fourth section concludes the paper with a summary and discussion.

## 2. Mesoscopic LBM Nonequilibrium Moments

In 1872, the renowned physicist Ludwig Boltzmann, building upon the existing kinetic theory of gases, formulated the Boltzmann equation applicable across diverse flow domains and introduced the Maxwell-Boltzmann distribution. This marked a significant advancement in the theory of gas kinetics. The specific form of the Boltzmann equation is:

(1) $$\frac{\partial f}{\partial t}+\xi \cdot \nabla_{x}f+a\cdot \nabla_{x}f=Q(f,f_{*})$$

The velocity distribution function f(u,x,t) is a function of the velocity vector u, spatial position vector x, and time t. Here, a represents the particle’s acceleration, and $Q(f,f_{*})$ accounts for the changes in the distribution function due to molecular collisions. The form is quite complex. The high-dimensional nonlinear integro-differential equation structure makes it challenging to provide an analytical solution to the Boltzmann equation from a theoretical perspective. Therefore, numerical simulation methods are commonly used as auxiliary means to solve the Boltzmann equation. In 1954, Prabhu Bhatnagar, Eugene Gross, and Max Krook proposed the BGK operator named after them [17], which, while ensuring mathematical and physical properties, discretizes the velocity space to simplify the solving process. The specific form of the BGK collision operator is: $\Omega (f)=-\frac{1}{\tau_{f}}\left(f-f^{\mathrm{eq}}\right)$. The symbol $\tau_{f}$ represents the relaxation time for density, and $f^{\mathrm{eq}}$ is the equilibrium state of the distribution function. Subsequently, the modern LBM has seen significant development by directly discretizing the particle velocity space in the Boltzmann-BGK equation. LBM is composed of a discrete velocity set, lattice structure, and evolution equations, namely:

(2) $$f(x+c_{\alpha }\delta t,t+\delta t)-f(x,t)=\Omega (f)$$

Here, x represents the points on the lattice, $c_{\alpha }$ is the discrete velocity set of fluid particles, and δt is the discrete time step.

(3) $$f^{\mathrm{eq}}=\omega_{\alpha }\rho \left(1+\frac{c_{\alpha }\cdot u}{c_{s}^{2}}+\frac{{\left(c_{\alpha }\cdot u\right)}^{2}}{2c_{s}^{4}}-\frac{u^{2}}{2c_{s}^{2}}\right)$$

Here, $\omega_{\alpha }$ represents the weighting coefficients, and $c_{s}$ denotes the speed of sound. Taking the D2Q9 discrete velocity model [4] as an example, the discrete velocities and their corresponding weighting coefficients are: $c_{\alpha }$ = $\left[\begin{array}{l} 0,1,0,-1,0,1,-1,-1,1\\ 0,0,1,0,-1,1,1,-1,-1 \end{array}\right]$, $\omega_{0}=\frac{4}{9}$, $\omega_{1-4}=\frac{1}{9}$, $\omega_{5-8}=\frac{1}{36}$. By migrating and colliding the particle points in the equation, the distribution function can be continuously updated, allowing for the simulation of the flow field.

### 2.1. Compressible LBM Model

The research methodology for nonequilibrium effects involves extracting nonequilibrium moments through LBM and establishing the physical significance of nonequilibrium effects based on their relationship with macroscopic equations. Therefore, this paper begins with compressible LBM models.

Currently, there are various compressible LBM models [16,17,18,20,22,32,33,34,35,39,40] capable of recovering the complete Navier-Stokes equations. The extraction of nonequilibrium moments is not influenced by the differences in LBM models, as long as they solve the Boltzmann-BGK equation or MRT-Boltzmann equation. These equations enable the acquisition of the required nonequilibrium moments. This paper takes the example of a double-distribution-function (DDF) LBM model [20] for illustration. The double-distribution function LBM model describes the evolution of density and total energy using two distribution functions:

(4) $$\frac{\partial f_{\alpha }}{\partial t}+\left(e_{\alpha }\cdot \nabla \right)f_{\alpha }=-\frac{1}{\tau_{f}}\left(f_{\alpha }-f_{\alpha }^{eq}\right)$$

(5) $$\frac{\partial h_{\alpha }}{\partial t}+\left(e_{\alpha }\cdot \nabla \right)h_{\alpha }=-\frac{1}{\tau_{h}}\left(h_{\alpha }-h_{\alpha }^{eq}\right)+\frac{1}{\tau_{hf}}\left(e_{\alpha }\cdot u-\frac{u^{2}}{2}\right)\left(f_{\alpha }-f_{\alpha }^{eq}\right)$$

In the equation, $f_{\alpha }^{eq}$ represents the discrete equilibrium distribution function for density, describing the molecular velocity distribution of the local equilibrium state. $f_{\alpha }$ represents the current state velocity distribution function. $h_{\alpha }^{eq}$ and $h_{\alpha }$ are discrete equilibrium distribution functions for total energy, corresponding to the equilibrium and current state distribution functions, respectively. Subscript α denotes the discrete velocity direction index, $e_{\alpha }$ is the discrete lattice velocity vector, and u is the macroscopic velocity vector. $\tau_{f}$ and $\tau_{h}$ are the relaxation times associated with $f_{\alpha }$ and $h_{\alpha }$ respectively. The relationship with $\tau_{hf}$ is: $1/\tau_{hf}=1/\tau_{h}-1/\tau_{f}$.

A crucial element in constructing an LBM model is determining the expression for the discrete equilibrium distribution functions. For compressible Navier-Stokes physical accuracy, $f_{\alpha }^{eq}$ and $h_{\alpha }^{eq}$ must satisfy the following seven conservation relations:

(6) $$M_{0}\left(f_{\alpha }^{eq}\right)=\sum_{\alpha }f_{\alpha }^{eq}=\rho$$

(7) $$M_{1,i}\left(f_{\alpha }^{eq}\right)=\sum_{\alpha }f_{\alpha }^{eq}e_{\alpha i}=\rho u_{i}$$

(8) $$M_{2,ij}\left(f_{\alpha }^{eq}\right)=\sum_{\alpha }f_{\alpha }^{eq}e_{\alpha i}e_{\alpha j}=\rho u_{i}u_{j}+p\delta_{ij}$$

(9) $$M_{3,ijk}\left(f_{\alpha }^{eq}\right)=\sum_{\alpha }f_{\alpha }^{eq}e_{\alpha i}e_{\alpha j}e_{\alpha k}=\rho u_{i}u_{j}u_{k}+p\left(u_{k}\delta_{ij}+u_{j}\delta_{ik}+u_{i}\delta_{jk}\right)$$

(10) $$M_{0}\left(h_{\alpha }^{eq}\right)=\sum_{\alpha }h_{\alpha }^{eq}=\rho E$$

(11) $$M_{1,i}\left(h_{\alpha }^{eq}\right)=\sum_{\alpha }h_{\alpha }^{eq}e_{\alpha i}=\left(\rho E+p\right)u_{i}$$

(12) $$M_{2,ij}\left(h_{\alpha }^{eq}\right)=\sum_{\alpha }h_{\alpha }^{eq}e_{\alpha i}e_{\alpha j}=\left(\rho E+2p\right)u_{i}u_{j}+p\left(E+RT\right)\delta_{ij}$$

In the equations, the subscripts I, j, and k refer to the x, y, and z-direction components. $E=\left(bRT+u^{2}\right)/2$ represents the total energy. T is the temperature. The relationship between b and the specific heat ratio γ is denoted as γ=(b+2)/b. In the middle of the equations are the moments of the equilibrium distribution function, representing the macroscopic expressions on the right side. On the left side, the symbols correspond to each moment, and the numerical subscript indicates the number of discrete velocities multiplied in the moment.

Clearly, there is not a unique set of $f_{\alpha }^{eq}$ and $h_{\alpha }^{eq}$ that satisfies the relationships (6)–(12). As long as these seven relationships are satisfied, an LBM model with physical accuracy for compressible Navier-Stokes equations can be obtained. The numerical characteristics of different $f_{\alpha }^{eq}$ and $h_{\alpha }^{eq}$ values may impact the numerical stability of the LBM model for solving compressible flows. However, as this is not the focus of this paper, it will not be discussed further here.

### 2.2. Nonequilibrium Effects

By multiplying both sides of Equation (4) by 1 and $e_{\alpha }$, respectively, and taking moments, then applying Equation (6) to Equation (8), we can obtain:

(13) $$\frac{\partial \rho }{\partial t}+\nabla \cdot \left(\rho u\right)=0$$

(14) $$\frac{\partial \left(\rho u\right)}{\partial t}+\nabla \cdot \left(\rho uu\right)=-\nabla p-\nabla \cdot \lambda_{f,2}$$

Similarly, by taking moments of Equation (5) on both sides and utilizing the relations in Equations (10) and (11), we can obtain:

(15) $$\frac{\partial \left(\rho E\right)}{\partial t}+\nabla \cdot \left(\rho Eu\right)=-\nabla \cdot \left(pu\right)-\nabla \cdot \lambda_{h,1}$$

where,

(16) $$\lambda_{f,2,ij}=\sum_{\alpha }\left(f_{\alpha }-f_{\alpha }^{eq}\right)e_{\alpha i}e_{\alpha j}$$

(17) $$\lambda_{h,1,i}=\sum_{\alpha }\left(h_{\alpha }-h_{\alpha }^{eq}\right)e_{\alpha i}$$

$\lambda_{f,2}$ can be regarded as the nonequilibrium moment corresponding to Equation (8), representing a nonequilibrium effect at that physical level. Similarly, $\lambda_{h,1}$ can be considered as the nonequilibrium moment corresponding to Equation (11). By comparing with the compressible Navier-Stokes equations, it can be observed that Equation (13) to Equation (14) correspond to the conservation equations for mass, momentum, and energy, respectively. In Equation (16), the nonequilibrium moment $\lambda_{f,2}$ is related to the viscous stress term Π in the momentum equation as follows:

(18) $$\lambda_{f,2}=-\Pi$$

And the nonequilibrium moment $\lambda_{h,1}$ in Equation (17) is related to the heat flux term $j_{q}$ as follows:

(19) $$\lambda_{h,1}=j_{q}-u\cdot \Pi$$

**Figure 1:**
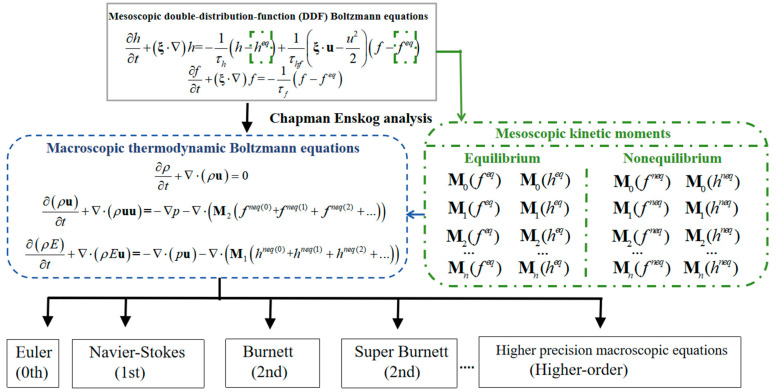
Schematic representation of the (non)equilibrium kinetic moment and its relation with the macroscopic thermohydrodynamic equations.

The paper provides a diagram (Figure 1) illustrating the relationship between the macroscopic fluid control equations at the Navier-Stokes level and the (non)equilibrium moments in the Boltzmann-BGK equation.

In fact, the validity of Equations (18) and (19) is subject to certain conditions. We derived these conclusions by comparing with the compressible Navier-Stokes equations. If we compare Equation (13) to Equation (15) with the Euler equations, we find that both nonequilibrium moments, $\lambda_{f,2}$ and $\lambda_{h,1}$, are equal to 0. Why does this happen? From Equations (16) and (17), it is evident that the values of nonequilibrium moments $\lambda_{f,2}$ and $\lambda_{h,1}$ depend on the accuracy of the current distribution functions $f_{\alpha }$ and $h_{\alpha }$. In Chapman-Enskog analysis, $f_{\alpha }$ can be expressed as:

(20) $$f_{\alpha }=f_{\alpha }^{eq}+\varepsilon f_{\alpha }^{\left(1\right)}+\varepsilon^{2}f_{\alpha }^{\left(2\right)}+\cdots$$

The symbol $\varepsilon^{n}f^{\left(n\right)}$ represents the nth order deviation of the velocity distribution function from the local equilibrium state distribution, where ε is a small quantity related to the Knudsen number. A smaller value of ε corresponds to the system being closer to the local equilibrium state. If the nonequilibrium term of $f_{\alpha }$ is neglected, i.e., $f_{\alpha }=f_{\alpha }^{eq}$, then the physical accuracy level of the LBM model is the same as that of the Euler equations. In this case, the fluid control equations do not consider the deviation of the fluid system from equilibrium. If only the first-order nonequilibrium term is considered, i.e., $f_{\alpha }=f_{\alpha }^{eq}+\varepsilon f_{\alpha }^{\left(1\right)}$, then the physical accuracy level of the LBM model is the same as that of the Navier-Stokes equations. In this case, nonequilibrium moments $\lambda_{f,2}$ and $\lambda_{h,1}$ are not equal to 0, and they represent the nonequilibrium effect-related terms in the fluid control equations. At this physical level, the contribution of nonequilibrium effects in the fluid control equations is demonstrated as shown in Equations (18) and (19). If the nonequilibrium term of $f_{\alpha }$ is retained to the second order or even higher, it is possible to derive fluid control equations with higher physical accuracy, such as the Burnett equations, super-Burnett equations, etc. [41]. In this process, the form of Equation (13) to Equation (15) does not change; what differs is the expression of nonequilibrium moments $\lambda_{f,2}$ and $\lambda_{h,1}$. Therefore, Equation (13) to Equation (15) can be considered as the framework of macroscopic fluid control equations, and macroscopic fluid control equations can be obtained based on the accuracy of $\lambda_{f,2}$ and $\lambda_{h,1}$.

From this, it is evident that nonequilibrium moments are crucial in fluid systems. For instance, in high-altitude rarefied gas fluid systems, the Navier-Stokes equations are not applicable. This is generally attributed to the derivation of the Navier-Stokes equations under the assumption of continuity. Alternatively, we can explain this from the perspective of nonequilibrium effects: the molecular motion nonequilibrium effects in rarefied gas systems are significant, and the Navier-Stokes equations only consider the first-order nonequilibrium effects of molecular motion, making it challenging to meet the required physical accuracy for nonequilibrium effects. In contrast, the Burnett equations, incorporating higher-order nonequilibrium moments, provide a more accurate description of nonequilibrium effects in rarefied gas fluid systems. Therefore, in complex fluid systems, analyzing the distribution characteristics of nonequilibrium effects contributes to an enhanced understanding of the mechanisms underlying flow phenomena.

## 3. Research Methods for Nonequilibrium Effects

The preceding discussion highlighted the significance of nonequilibrium effects in fluid systems. This section will introduce the methodology for studying fluid systems using nonequilibrium effects. This approach was proposed and developed by Professor Xu’s research group [3,8,42], and we, inspired by their work, have introduced a method to understand and apply nonequilibrium effects from the perspective of molecular motion [43,44]. Here, we will primarily focus on the fundamental ideas of this method, the interpretation based on molecular motion kinematics, and the physical significance of the strength and direction of nonequilibrium effects. It should be noted that this section presents our understanding of nonequilibrium effects, which may slightly differ from Professor Xu’s research group’s perspective on the physical significance of nonequilibrium effects. Both interpretations are considered reasonable, with differences arising from varying perspectives on understanding nonequilibrium effects.

### 3.1. Equilibrium Kinetic Moment

Equations (15)–(18) and Equations (10)–(12) represent the equilibrium moments corresponding to the compressible Navier-Stokes level. Nonequilibrium moments are derived from equilibrium moments by taking moments of the nonequilibrium quantities in the distribution function. Therefore, let us initiate the discussion by focusing on equilibrium moments.

From Equations (15)–(18) and Equations (10)–(12), it can be observed that equilibrium moments can be divided into moments containing the mass density distribution function $f_{\alpha }^{eq}$ and moments containing the total energy density distribution function $h_{\alpha }^{eq}$. In Equation (7), the equilibrium moment is composed of the product of $f_{\alpha }^{eq}$ and $e_{\alpha i}$. It is known that mass multiplied by velocity is momentum at the macroscopic level. Therefore, $\sum_{\alpha }f_{\alpha }^{eq}e_{\alpha i}$ corresponds to the macroscopic momentum density. In Equation (8), $\sum_{\alpha }f_{\alpha }^{eq}e_{\alpha i}e_{\alpha j}$, when i=j, represents the translational kinetic energy density. Looking back at the history of physics, momentum mv was initially defined to describe the motion state of an object in the direction of velocity v. Subsequently, the kinetic energy $m{\left|v\right|}^{2}/2$ was introduced to characterize the motion state of an object with thermodynamic information. If an object is only moving in i direction, its momentum and kinetic energy in that direction are $mv_{i}$ and $mv_{i}^{2}/2$, respectively. The essential mathematical difference between the two is that kinetic energy $mv_{i}^{2}/2$ involves an additional multiplication by velocity compared to momentum $mv_{i}$. “$\sum_{\alpha }f_{\alpha }^{eq}e_{\alpha i}e_{\alpha j}$” can be understood as an equilibrium moment at the same physical level as kinetic energy $mv_{i}^{2}/2$. In Equation (9), the equilibrium moment $\sum_{\alpha }f_{\alpha }^{eq}e_{\alpha i}e_{\alpha j}e_{\alpha k}$ involves an additional multiplication by velocity $e_{\alpha i}$ compared to $\sum_{\alpha }f_{\alpha }^{eq}e_{\alpha i}e_{\alpha j}$. We can easily associate this with a physical quantity: the kinetic energy of an object in motion along i direction multiplied by velocity, i.e., $mv_{i}^{3}$. Although such a quantity is rarely used, the analysis above reveals that it represents a macroscopic quantity describing the motion of an object, providing information distinct from momentum or kinetic energy. $\sum_{\alpha }f_{\alpha }^{eq}e_{\alpha i}e_{\alpha j}e_{\alpha k}$ can be understood as an equilibrium moment at its own physical level. It is important to note that this discussion is not an accurate physical definition of equilibrium moments but rather a means to comprehend their physical significance through macroscopic momentum and kinetic energy.

Thus, equilibrium moments can be defined within a generalized moment framework, which we refer to as the kinetic moment:

(21) $$M_{kinetic}=\sum_{\alpha }\Psi_{\alpha }$$

Here, $\Psi_{\alpha }=\phi_{\alpha }\xi_{\alpha i}\xi_{\alpha j}\cdots$ is a motion state variable composed of a physical property $\phi_{\alpha }$ and several motion multipliers $\xi_{\alpha }$. In Equations (6)–(9), $\phi_{\alpha }$ is the mass density equilibrium distribution function $f_{\alpha }^{eq}$, and in Equations (10)–(12), $\phi_{\alpha }$ is the total energy density equilibrium distribution function $h_{\alpha }^{eq}$. $\xi_{\alpha }$ is the characteristic velocity at the scale of $\phi_{\alpha }$, which in Equations (6)–(9) and Equations (10)–(12) corresponds to the discrete molecular velocity $e_{\alpha }$. Kinetic moments are moments of a physical property $\phi_{\alpha }$ multiplied by motion multipliers $\xi_{\alpha }$. Kinetic moments with more motion multipliers, denoted as $\xi_{\alpha }$, contain more information about motion, and the influence of motion on the physical property is greater. Thus, a quantity such as $\sum_{\alpha }f_{\alpha }^{eq}e_{\alpha i}e_{\alpha j}$ contains more “motion action” compared to $\sum_{\alpha }f_{\alpha }^{eq}e_{\alpha i}$, providing a better understanding of the physical meaning of higher-order moments. This framework allows for a molecular-level understanding of the physical meanings of equilibrium and nonequilibrium moments. For macroscopic quantities, a similar approach using state variables $\Psi_{\alpha }$ can help in understanding their physical meanings. For instance, considering $\rho u_{i}u_{j}$ in Equation (8), where density ρ is a physical property, and $u_{i}$ and $u_{j}$ are two motion multipliers. Building on this, we can better understand the physical meaning of $\rho u_{i}u_{j}u_{k}$ in Equation (9).

Table 1 and Table 2 present the physical meanings of mass density and total energy density equilibrium moments based on the kinetic moment concept. It is important to note the following:

1. The discussion here focuses on physical meanings rather than precise definitions.
2. The units of each equilibrium moment are provided in the tables to aid in understanding the physical meanings of higher-order moments.
3. A state variable with n motion multipliers is referred to as an n-order state. Higher-order states contain stronger information about material “motion action”.
4. $f_{\alpha }^{eq}e_{\alpha i}$ represents the i-directional component of the momentum density for the α component in the equilibrium state, and $f_{\alpha }^{eq}e_{\alpha i}e_{\alpha j}$ is the i-directional component of the translational kinetic energy density for the α component in the equilibrium state.

### 3.2. Nonequilibrium Kinetic Moment

Substituting the variables $f_{\alpha }^{eq}$ and $h_{\alpha }^{eq}$ in Equations (6)–(9) and (13) with $\left(f_{\alpha }-f_{\alpha }^{eq}\right)$ and $\left(h_{\alpha }-h_{\alpha }^{eq}\right)$, respectively, yields the nonequilibrium moments. Utilizing the above-discussed physical meanings of equilibrium kinetic moments, the physical interpretation of the nonequilibrium kinetic moments can naturally be derived, representing the local nonequilibrium state of kinetic moments in Table 1 and Table 2.

In this study, we take the kinetic moments at the Navier-Stokes level as an example for discussion. Since mass, momentum, and energy are conserved in fluid systems, $\lambda_{f,0}=\lambda_{f,1}=\lambda_{h,0}=0$. The nonequilibrium moments of interest are $\lambda_{f,2}$, $\lambda_{f,3}$, $\lambda_{h,1}$, and $\lambda_{h,2}$, where each has two terms related to mass density and total energy density. With Equations (18) and (19), and the corresponding macroscopic expressions known for $\lambda_{f,2}$ and $\lambda_{h,1}$, the physical interpretations of the nonequilibrium moments corresponding to viscous stress and heat flux can be obtained. However, higher-order nonequilibrium moments $\lambda_{f,3}$ and $\lambda_{h,2}$ are not present in the macroscopic fluid control equations (Equations (13)–(15)).

In the context of Chapman-Enskog analysis, by only retaining the first-order nonequilibrium terms, the relationship between variables $f_{\alpha }^{neq}$ and $f_{\alpha }^{eq}$ can be obtained:

(22) $$f_{\alpha }^{neq}=f_{\alpha }-f_{\alpha }^{eq}=-\tau_{f}\cdot (\frac{\partial f_{\alpha }^{eq}}{\partial t}+e_{\alpha }\cdot \nabla f_{\alpha }^{eq})$$

We employed this relationship to deduce the association between nonequilibrium moments and equilibrium moments [38]:

(23) $$\lambda_{f,n,\hat{i}}=-\tau_{f}\cdot \left[\frac{\partial M_{n,\hat{i}}\left(f_{\alpha }^{eq}\right)}{\partial t}+\nabla \cdot M_{n+1,\hat{i}\hat{j}}\left(f_{\alpha }^{eq}\right)\right]$$

(24) $$\lambda_{h,n,\hat{i}}=-\tau_{h}\cdot \left[\frac{\partial M_{n,\hat{i}}\left(h_{\alpha }^{eq}\right)}{\partial t}+\nabla \cdot M_{n+1,\hat{i}\hat{j}}\left(h_{\alpha }^{eq}\right)\right]+\frac{\tau_{h}}{\tau_{hf}}\cdot \left(u\cdot \lambda_{f,n+1,\hat{i}\hat{j}}-\frac{u^{2}}{2}\cdot \lambda_{f,n,\hat{i}}\right)$$

In the expressions, the subscript $\hat{i}$ corresponds to i, ij, or ijk, and the subscript $\hat{j}$ represents the vector component obtained by multiplying $e_{\alpha }$ and $f_{\alpha }^{eq}$. Equations (23) and (24) are derived based on Chapman-Enskog analysis to define nonequilibrium effects at the Navier-Stokes level, leading to expressions for the nonequilibrium moments $\lambda_{f,2}$, $\lambda_{f,3}$, $\lambda_{h,1}$, and $\lambda_{h,2}$. Here, the subscripts f and h respectively denote nonequilibrium effects related to mass and total energy, and the numbers 1 and 2 represent the corresponding orders. It is noteworthy that the calculation of nonequilibrium moments requires the use of higher-order equilibrium moments. For $\lambda_{f,2}$ and $\lambda_{h,1}$, the corresponding equilibrium moments at the Navier-Stokes level involve their respective higher-order $M_{3}\left(f_{\alpha }^{eq}\right)$ and $M_{2}\left(h_{\alpha }^{eq}\right)$. However, $M_{4}\left(f_{\alpha }^{eq}\right)$ and $M_{3}\left(h_{\alpha }^{eq}\right)$, which are one order higher than $\lambda_{f,3}$ and $\lambda_{h,2}$, respectively, are not included in Equations (6)–(9) and (10)–(12). Therefore, to calculate $\lambda_{f,3}$ and $\lambda_{h,2}$ using Equations (23) and (24), the expressions for $M_{4}\left(f_{\alpha }^{eq}\right)$ and $M_{3}\left(h_{\alpha }^{eq}\right)$ are needed, which can be found in the literature that has considered LBM models beyond the Navier-Stokes level [36]. Furthermore, Equations (23) and (24) allow for an analysis of the strength and directional characteristics of nonequilibrium effects. It is worth noting that the nonequilibrium moments characterizing system nonequilibrium effects, as described above, encompass information about both macroscopic quantities and particle fluctuations. In the literature [43,44,45], nonequilibrium moments in this form have been utilized to represent nonequilibrium effects. Additionally, in the literature [3,17,28,29,30,31,46,47], the macroscopic velocity’s influence has been subtracted during the moment calculation processes, as shown in Equations (7)–(9) and (11)–(12).

(25) $$M_{1,i}^{*}\left(f_{\alpha }^{eq}\right)=\sum_{\alpha }f_{\alpha }^{eq}\left(e_{\alpha i}-u\right)$$

(26) $$M_{2,ij}^{*}\left(f_{\alpha }^{eq}\right)=\sum_{\alpha }f_{\alpha }^{eq}\left(e_{\alpha i}-u\right)\left(e_{\alpha j}-u\right)$$

(27) $$M_{3,ijk}^{*}\left(f_{\alpha }^{eq}\right)=\sum_{\alpha }f_{\alpha }^{eq}\left(e_{\alpha i}-u\right)\left(e_{\alpha j}-u\right)\left(e_{\alpha k}-u\right)$$

(28) $$M_{1,i}^{*}\left(h_{\alpha }^{eq}\right)=\sum_{\alpha }h_{\alpha }^{eq}\left(e_{\alpha i}-u\right)$$

(29) $$M_{2,ij}^{*}\left(h_{\alpha }^{eq}\right)=\sum_{\alpha }h_{\alpha }^{eq}\left(e_{\alpha i}-u\right)\left(e_{\alpha j}-u\right)$$

In the paper, nonequilibrium effects associated with the distribution function $f_{\alpha }^{eq}$ are referred to as non-organized momentum flux (NOMF), while those related to the $h_{\alpha }^{eq}$ are termed non-organized energy flux (NOEF). It is important to note that symbols used may vary across different sources.

However, regardless of the chosen representation, gaining a clear understanding of the magnitude and directional implications of non-equilibrium moments remains challenging and may require further investigation, especially to comprehend their physical significance. Therefore, this paper introduces an approach, as expressed in Equations (23) and (24), to discuss the physical implications of nonequilibrium effects from a molecular motion perspective.

**Figure 2:**
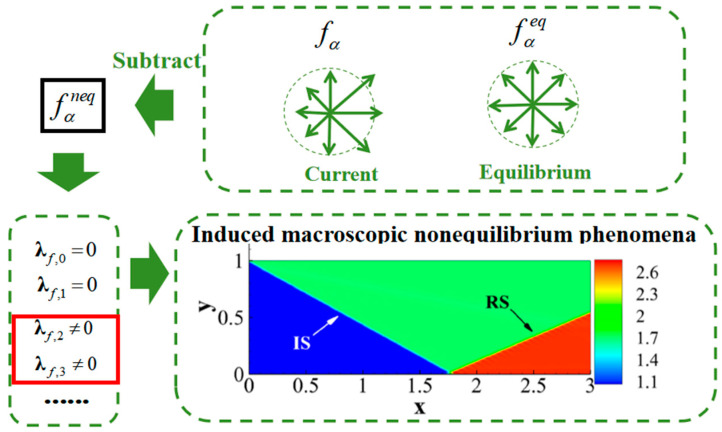
Schematic representation of the nonequilibrium effects derived from the molecular distribution function.

Returning to the fundamentals of the Boltzmann-BGK equation and MRT-Boltzmann equation, the description of fluid flow involves the local nonequilibrium state $\left(f_{\alpha }-f_{\alpha }^{eq}\right)$ of the molecular distribution function. Here, the equilibrium distribution function $f_{\alpha }^{eq}$ represents the local equilibrium state of molecular motion, while the current distribution function $f_{\alpha }$ represents the actual state of molecular motion. In regions where the fluid system exhibits nonequilibrium behavior, the distributions of these two functions do not align. But due to mass and momentum conservation, $\lambda_{f,0}$ as well as $\lambda_{f,1}$ are zero. Drawing from the principles of kinetic theory, it is understood that $\lambda_{f,1}$ has a single motion multiplier, while $\lambda_{f,2}$ and $\lambda_{f,3}$ have more motion multipliers, indicating a stronger “motion effect”. This effect amplifies the nonequilibrium effects of $\left(f_{\alpha }-f_{\alpha }^{eq}\right)$, resulting in nonzero values for $\lambda_{f,2}$ and $\lambda_{f,3}$. Figure 2 provides a somewhat idealized example to illustrate this concept. In the schematic diagram, the velocity distributions of $f_{\alpha }$ and $f_{\alpha }^{eq}$ differ, and under the amplifying effect of motion multipliers, the components of $\lambda_{f,2}$ and $\lambda_{f,3}$ in the x-direction are both greater than 0. For locations where the macroscopic flow velocity $u_{x}>0$, positive components of the nonequilibrium moments in the x-direction indicate that at these positions, the current-state kinetic moments deviate from the local equilibrium-state kinetic moments along the positive x-direction. Both moments reflect the motion state of molecular clusters, with the former exhibiting a stronger motion state. If the nonequilibrium moments are negative, different scenarios need to be considered, such as:

1. The current-state kinetic moments remain positive, indicating a weaker motion compared to the kinetic moments of the local equilibrium state.
2. The current-state kinetic moments are negative, with an absolute value smaller than those of the local equilibrium kinetic moments. In this case, not only is the motion weaker in the former compared to the latter, but their directions of motion also differ.
3. The current-state kinetic moments are negative, with an absolute value greater than those of the local equilibrium kinetic moments. In this scenario, the motion in the former is stronger than in the latter, and their directions of motion are distinct.

In summary, the physical significance of nonequilibrium effects can be explained as follows:

1. Nonequilibrium effects reflect the extent to which molecular motion deviates from local equilibrium states, mapped onto the corresponding physical levels of mass and total energy. This deviation can be understood as a kind of “instability” of the local system relative to equilibrium states.
2. The sign of nonequilibrium effects indicates the direction in which molecular motion deviates from its local equilibrium state.
3. The magnitude of nonequilibrium effects reflects the local “instability” level of molecular motion at the corresponding hierarchy.
4. With this perspective, nonequilibrium behavior can be interpreted in a more intuitive manner. The nonequilibrium effects research method discussed in this paper is not limited to specific LBM models. At the Navier-Stokes level, any model that satisfies the moment relations in Equations (2) and (3) can be applied.

## 4. Nonequilibrium Effects Study on Shock-Related Problems

**Figure 3:**
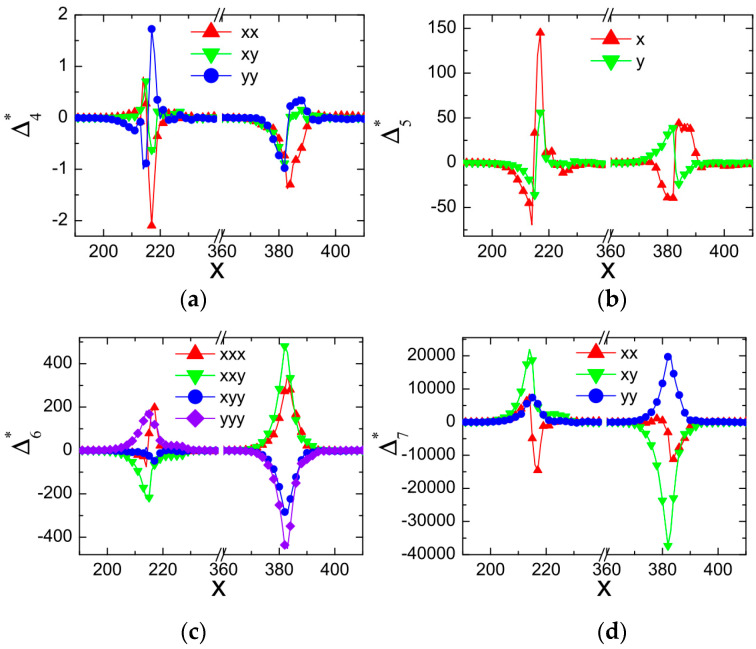
Nonequilibrium effect distribution along the horizontal line in a regular reflection shock wave [17]: (**a**) $\Delta_{4}^{*}$ (**b**) $\Delta_{5}^{*}$ (**c**) $\Delta_{6}^{*}$ (**d**) $\Delta_{7}^{*}$.

A shock wave is one of the most typical flow phenomena in supersonic fluid dynamics, widely present in the internal and external flows of aerospace equipment such as supersonic aircraft, missiles, and rockets. In 2013, Gan et al. [17] applied the nonequilibrium effects method to the study of two-dimensional shock wave reflection problems. They preliminarily demonstrated the distribution characteristics of various nonequilibrium moments at the incident and reflected shock waves. As shown in Figure 3, different intensities and directions of nonequilibrium effects are observed at the shock wave locations. In the figure, the variable $\Delta_{n}^{*}$ represents the nonequilibrium effects of the nth order. It is evident that utilizing nonequilibrium effects to study such flow-discontinuity issues, such as shock waves, provides a different perspective on flow mechanics compared to traditional macroscopic variables such as density and temperature.

In recent years, a series of research studies have been conducted on the nonequilibrium effects in flow-discontinuity problems, such as shock waves and detonation waves. The following provides an overview of the research progress in this area. It is important to note that in the following research progress, the double-distribution-function LBM model used in references [38,43] is different from the research conducted by Professor Xu’s group, which is based on the Discrete Boltzmann Method (DBM) [3,17,28,29,30,31]. The primary objective of this paper is to comprehensively review the advancements in research on the nonequilibrium effects of shock waves. The details of the theoretical method, simulation configuration, and numerical verification of the corresponding cases can be found in the relevant literature for readers’ reference, thus avoiding repetition here.

### 4.1. Shock Wave

The one-dimensional Riemann problem is a typical one-dimensional shock wave problem, and its evolution involves shock waves, expansion waves, and contact discontinuities, encompassing various flow characteristics of waves. Moreover, the one-dimensional nature of the problem eliminates the interference from the two-dimensional flow direction, simplifying the complexity of analyzing nonequilibrium effects and facilitating the examination of the shock wave’s nonequilibrium behavior.

Recently, we conducted research on the nonequilibrium effects of the one-dimensional Riemann problem [43]. The initial conditions are specified as follows:

$$\left(\rho ,u_{x},p\right)=\left\{ \begin{array}{l} \left(1.0,0,1.0\right),0\leq x/L_{0}\leq 0.5,\\ \left(0.125,0,0.1\right),0.5<x/L_{0}\leq 1. \end{array}\right.$$

**Figure 4:**
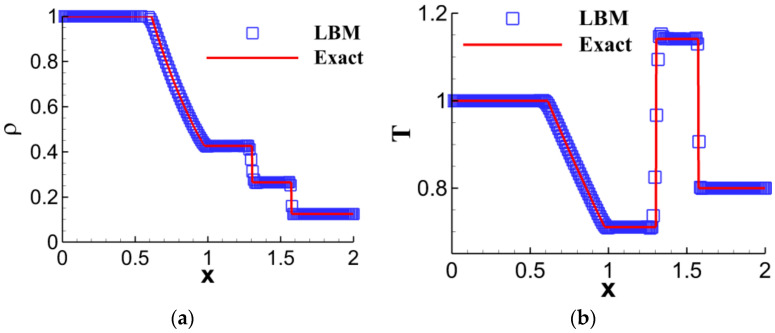
Macroscopic quantity distribution of the Sod shock tube at t = 0.1644t_0_ [48]: (**a**) Density (**b**) Temperature.

ρ represents density, $u_{x}$ denotes the transverse velocity, p stands for pressure, and $L_{0}$ represents the reference length. The traditional approach involves describing the shock wave evolution process of the Riemann problem using macroscopic quantities. Figure 4 shows the density and temperature distributions at the moment t = 0.1644t_0_ for the LBM solution of the Sod shock tube problem. The results by LBM are compared with the analytical solutions, which are in good agreement. Among these, $t_{0}=L_{0}/u_{0}$ represents the dimensionless reference time. Macroscopic quantities exhibit significant gradient changes at the location of the expansion wave, while flow discontinuities are observed at the shock wave and contact discontinuity locations.

**Figure 5:**
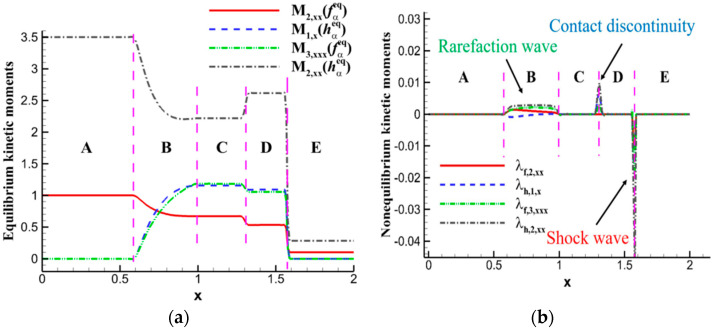
Moments distribution of the Sod shock tube at t = 0.1644t_0_ [48]: (**a**) Equilibrium moments (**b**) Nonequilibrium moments.

Figure 5 illustrates the distribution of equilibrium moments and nonequilibrium moments in the flow field of the Sod shock tube. Since $\lambda_{f,0}=\lambda_{f,1}=\lambda_{h,0}=0$, only the nonzero equilibrium moments and nonequilibrium moments are considered. For analytical purposes, the flow field is divided into five regions (A–E) based on the shock wave, contact discontinuity, and expansion wave. Region B corresponds to the expansion wave, the junction between regions C and D represents the contact discontinuity, and the junction between regions D and E represents the shock wave. The distribution characteristics of equilibrium moments are quite similar to macroscopic quantities, showing flow discontinuities at the shock wave and contact discontinuity locations, while the expansion wave exhibits noticeable fluctuations, and other positions remain at their corresponding constants. The distribution characteristics of nonequilibrium moments, however, differ: the absolute values significantly increase at the shock wave and contact discontinuity, forming peaks or valleys; there is a slight increase in the absolute values at the expansion wave; and the values of nonequilibrium moments at other positions are zero. The results of nonequilibrium moment distribution indicate a strong molecular motion instability at the shock wave and contact discontinuity, reflecting a pronounced instability relative to local equilibrium states, with the former exhibiting significantly greater intensity than the latter. Moreover, the direction of nonequilibrium effects also shows distinct distribution characteristics at the shock wave, contact discontinuity, and expansion wave locations. For a detailed analysis of nonequilibrium effects, refer to the literature [48]. Additionally, verification of nonequilibrium moments is provided in Section III.D of the same reference [48].

**Figure 6:**
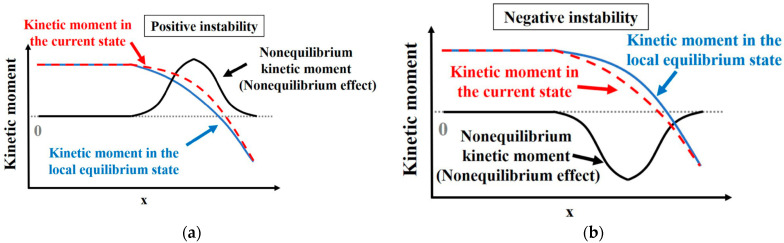
Schematic representation of nonequilibrium effects [48]. (**a**) Positive instability (**b**) Negative instability.

To further discuss the meaning of nonequilibrium effects, consider the schematic diagram in Figure 6. The red dashed line represents the kinetic moments of the current state, the blue solid line represents the kinetic moments of the local equilibrium state, and the black solid line represents the nonequilibrium effects; i.e., the difference between the corresponding current moments and local equilibrium moments. When the red line coincides with the blue line, the system’s current state is almost identical to the local equilibrium state, and the nonequilibrium effects along the black line are nearly zero. In cases where the red line is above the blue line, as shown in Figure 6a, the current moments of the system have larger values than the local equilibrium moments, and the nonequilibrium effects along the black line show positive values, reaching a peak where the difference between the two is greatest. The opposite occurs when the red line is below the blue line, as shown in Figure 6b. For ease of analysis, situations where nonequilibrium effects are positive are referred to as “positive instability”, and situations where nonequilibrium effects are negative are referred to as “negative instability”. This provides a definition for the “unstable” direction of molecular motion. It is important to note that when nonequilibrium effects are positive, there are roughly three cases: first, both the current moments and local equilibrium moments are positive, with the former having a larger absolute value than the latter; second, both are negative, with the absolute value of the current moments smaller than that of the local equilibrium moments; third, the current moments are positive, and local equilibrium moments are negative, with the absolute values not necessarily equal. Of course, there are also special cases where one of them is zero, which requires specific analysis depending on the circumstances.

**Figure 7:**
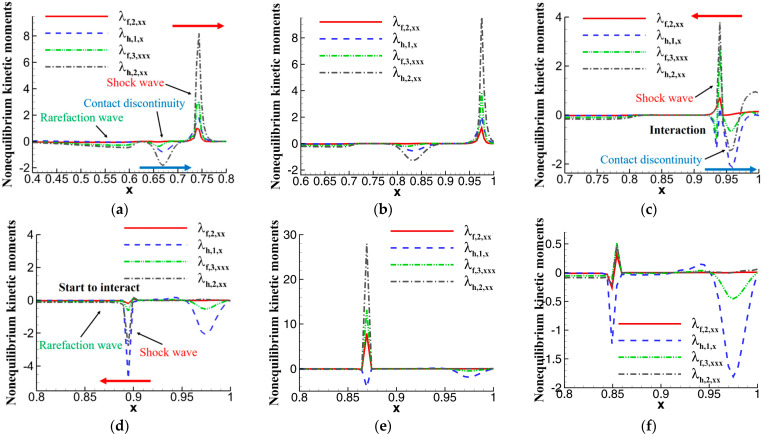

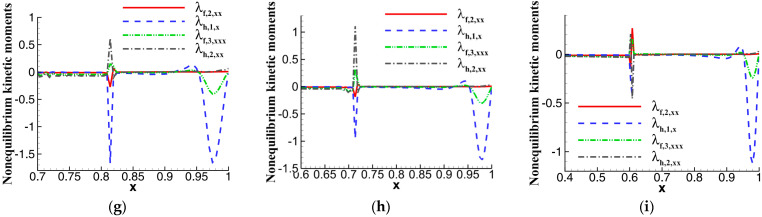
Evolution of nonequilibrium kinetic moments in the process of reflected shock in a shock tube [48]: (**a**) t = 0.1t_0_ (**b**) t = 0.2t_0_ (**c**) t = 0.3t_0_ (**d**) t = 0.45t_0_ (**e**) t = 0.5t_0_ (**f**) t = 0.53t_0_ (**g**) t = 0.6t_0_ (**h**) t = 0.8t_0_ (**i**) t = 1.0t_0_.

Based on this foundation, we conducted a one-dimensional unsteady simulation of the shock wave reflection case inside the shock tube presented by Daru and Tenaud [49]. The distribution of nonequilibrium moments at various time steps is shown in Figure 7. The flow field exhibits shock waves, contact discontinuities, and expansion waves. In the initial stages of flow evolution, all three exhibit a trend of moving to the right. The shock wave is in a relatively strong “positive instability” state, while the contact discontinuity and expansion wave are in a relatively weak “negative instability” state. When the shock wave reflects from the right wall at the beginning of the simulation and changes its direction to the left, it encounters the contact discontinuity moving to the right at t = 0.3. At this moment, due to the opposite “unstable” directions of the shock wave and the contact discontinuity, the nonequilibrium effects of the shock wave decrease, and the intensity of nonequilibrium effects of the contact discontinuity at the shock wave location also significantly decreases. This indicates that the interaction between shock waves and contact discontinuities can mutually suppress nonequilibrium effects in different directions. As they move apart in opposite directions, the separated shock wave is greatly influenced, and the nonequilibrium effects change from positive to negative, an unstable state. When the shock wave encounters the expansion wave, their interaction causes a strong rebound in the nonequilibrium effects of the shock wave, exhibiting a stronger “positive instability” state than the early stages of flow evolution. This suggests that under the interaction of shock waves with contact discontinuities or expansion waves, the instability of molecular motion significantly increases, and the state deviating from local equilibrium becomes extremely unstable. Subsequently, as the shock wave continues to move to the left and interacts with the expansion wave, the nonequilibrium effects of the shock wave become very weak, with an intensity smaller than that of the contact discontinuity. Thus, from the perspective of nonequilibrium effects in molecular motion, the interaction phenomena of shock waves, contact discontinuities, and expansion waves are elucidated.

Lin et al. conducted a study on the characteristics near a one-dimensional shock wave, focusing on the velocity distribution function and nonequilibrium intensity [28]. The initial state is set by the Hugoniot relation:

$$\left\{ \begin{array}{l} {\left(\rho ,u_{x},T,p\right)}_{left}=\left(2.667,1.479,1.688,4.5\right),0\leq x/L_{0}\leq 0.01,\\ {\left(\rho ,u_{x},T,p\right)}_{right}=\left(1,0,1,1\right),0.01<x/L_{0}\leq 1 \end{array}\right.$$

**Figure 8:**
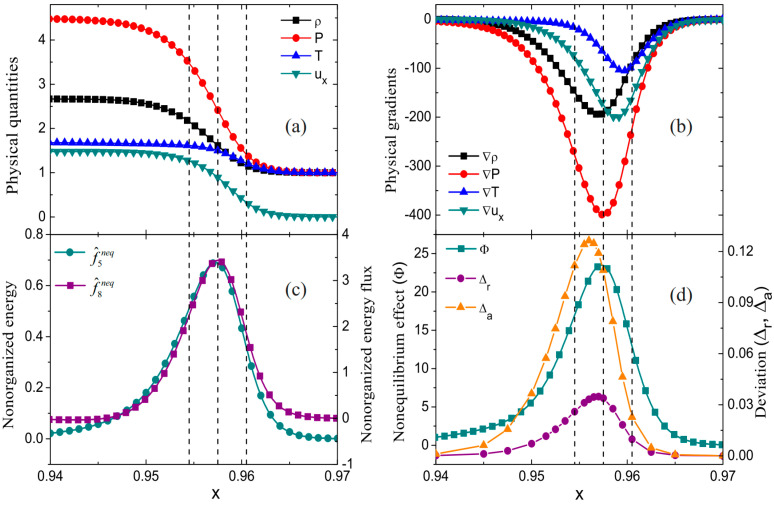
Profiles of [28]: (**a**) Macroscopic physical quantities (**b**) Physical gradients (**c**) Nonorganized energy and energy flux in the *x* direction (**d**) Nonequilibrium effect and deviation degrees near the wavefront.

**Figure 9:**
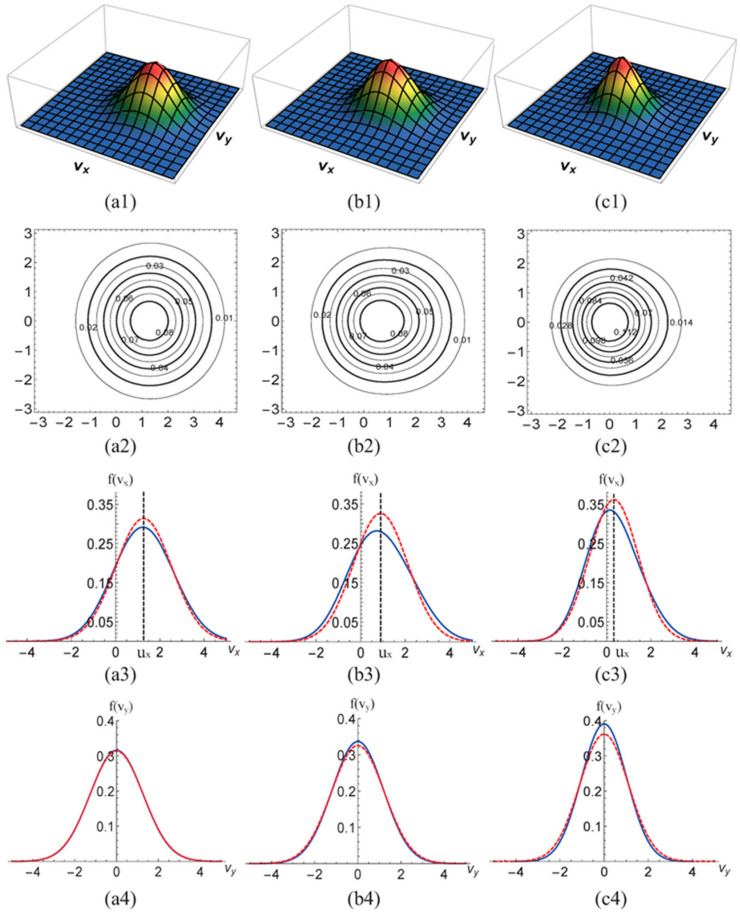
Profiles of velocity distribution functions near the wavefront [28]: (**a1**–**c1**) 3D velocity distribution functions (**a2**–**c2**) 2D velocity distribution functions (**a3**–**c3**,**a4**–**c4**) current state of the molecular velocity distribution function (blue line) and its corresponding local equilibrium state (red line).

Figure 8 illustrates the distribution of macroscopic quantities, macroscopic quantity gradients, partial nonequilibrium quantities, and nonequilibrium intensity near the shock wave. Locations where nonequilibrium effects increase are observed to correspond to regions with larger gradients of macroscopic quantities, such as velocity and temperature near the shock wave. Figure 9 shows the distribution characteristics of the velocity distribution function at three typical positions: ahead of the wave, within the wave, and behind the wave. From observation of the figure, the shape of the velocity distribution function peaks becomes more concentrated from behind the wave to ahead of the wave. This is due to the rapid changes in physical quantities as the shock wave passes through the medium. Meanwhile, in the two-dimensional distribution in the second row, an x-direction asymmetry between ahead of the wave and behind the wave is observed, indicating the presence of nonequilibrium effects.

**Figure 10:**
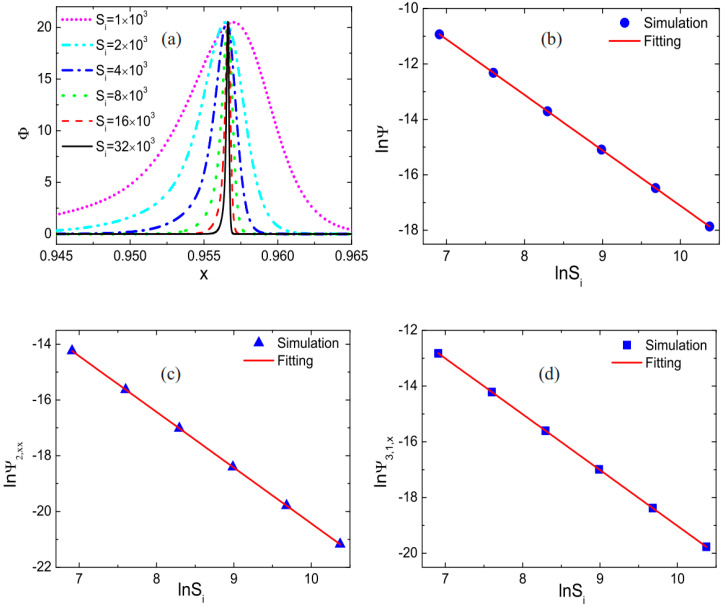
Relationship between the strength of nonequilibrium effects Φ and the natural logarithm of the relaxation frequency $S_{i}$ [28]: (**a**) Φ with various $S_{i}$ (**b**) Relationship between lnΨ and $\ln S_{i}$ (**c**) Relationship between $\ln\Psi_{2xx}$ and $\ln S_{i}$ (**d**) Relationship between $\ln\Psi_{31x}$ and $\ln S_{i}$.

Subsequently, they investigated the influence of fluid properties or parameters such as the relaxation frequency, the Mach number, thermal conductivity, viscosity, and the specific heat ratio on the strength of nonequilibrium effects near shock waves, further revealing the nonequilibrium behavior near shock waves. Figure 10 depicts the nonequilibrium effect strength at shock waves under different relaxation frequencies. As the relaxation frequency increases, the peak strength of nonequilibrium effects at shock waves remains almost unchanged, while the region of nonequilibrium effects decreases. This is because with an increase in relaxation frequency, the relaxation time for the system to approach local equilibrium decreases, naturally leading to a reduction in nonequilibrium effects near shock waves. A linear relationship between the strength of nonequilibrium effects and the natural logarithm of the relaxation frequency was observed through linear equation fitting. The impact of other fluid properties or parameters on the strength of nonequilibrium effects at shock waves can be found in reference [28].

**Figure 11:**
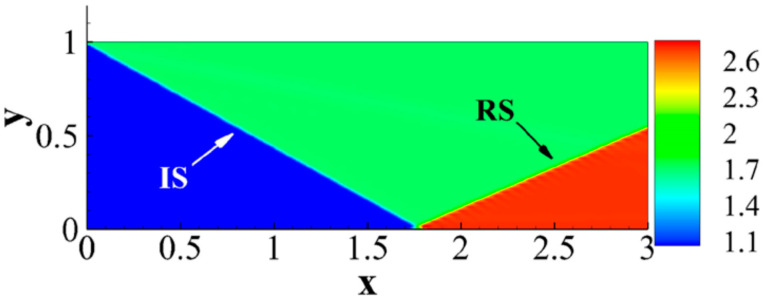
Distributions of density [44].

The two-dimensional flow is more universal than the one-dimensional counterpart. Figure 11 is the density distribution of two-dimensional regular reflected shock waves. The flow field in shock wave reflection includes incident and reflected shock waves. The inflow Mach number simulated is 2.9. Dirichlet conditions are applied on the left and upper boundaries as follows:

$\left(\rho ,u_{x},u_{y},p\right){|}_{left}=\left(1.0,2.9\sqrt{\gamma },0,1.0\right),\left(\rho ,u_{x},u_{y},p\right){|}_{top}=\left(1.69997,2.61934\sqrt{\gamma },-0.50633\sqrt{\gamma },2.139466\right)$, the specific heat ratio γ=1.4. By solving the unsteady shock wave reflection problem, the process involves the formation of moving curved shock waves, exhibiting richer shock wave characteristics. We conducted a study on the formation process of two-dimensional shock waves and the nonequilibrium behavior of the system reaching a steady state [43]. The following is a brief introduction using symbols $M_{f}^{\mathrm{neq}}$ and $M_{h}^{\mathrm{neq}}$ to represent nonequilibrium moments.

**Figure 12:**
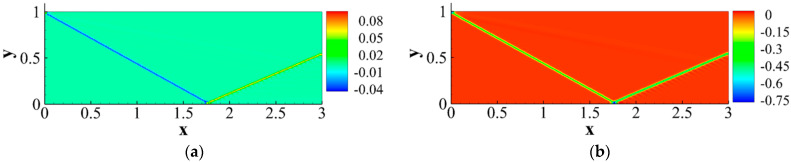
Distributions of nonequilibrium effects in a regular reflection shock wave [44]: (**a**) $M_{h1,y}^{\mathrm{neq}}$ (**b**) $M_{f3,xyy}^{\mathrm{neq}}$.

Figure 12 illustrates the distribution of two nonequilibrium moments, $M_{h1,y}^{\mathrm{neq}}$ and $M_{f3,xyy}^{\mathrm{neq}}$. Apart from the strong nonequilibrium effects near the shock waves, the rest of the spatial flow field exhibits almost zero nonequilibrium effects. This is consistent with the nonequilibrium behavior observed in one-dimensional shock wave flow. Interestingly, the characteristics of nonequilibrium effects for $M_{h1,y}^{\mathrm{neq}}$ and $M_{f3,xyy}^{\mathrm{neq}}$ at the incident and reflected shock waves differ. The former shows different “unstable” directions at these locations, while the latter maintains a consistent direction. This indicates that certain nonequilibrium effects manifest distinct characteristics at incident and reflected shock waves. Leveraging this feature might contribute to a better understanding of complex shock wave systems.

**Figure 13:**
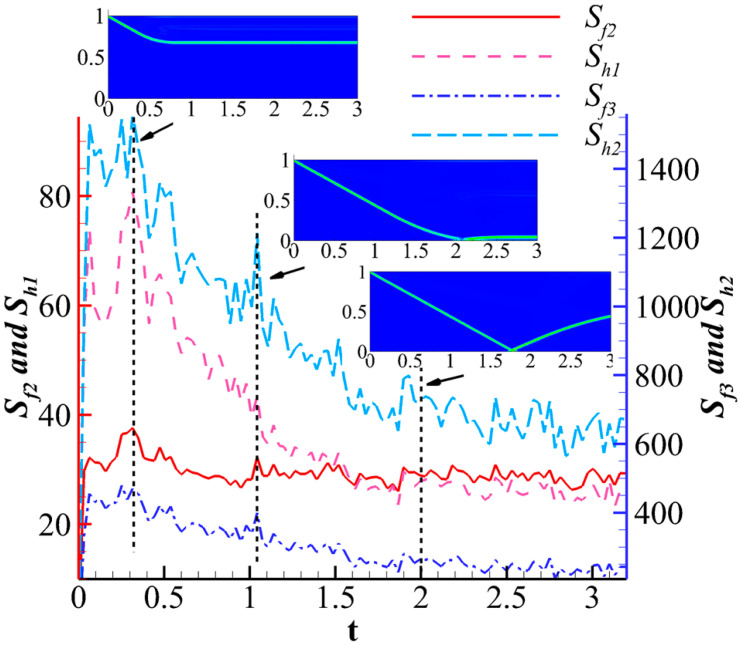
Strengths of nonequilibrium effects at different physical levels during the formation of a regular reflection shock wave [44].

Figure 13 illustrates the evolution of the system’s nonequilibrium effects during the shock wave reflection process. The definition of nonequilibrium effect intensity $S_{f2}$, $S_{f3}$, $S_{h1}$, $S_{h2}$ is:

(30) $$\begin{array}{l} S_{f2}=\int \sqrt{{\left(M_{f2,xx}^{neq}\right)}^{2}+{\left(M_{f2,yy}^{neq}\right)}^{2}+{\left(M_{f2,xy}^{neq}\right)}^{2}}dxdy\\ S_{h1}=\int \sqrt{{\left(M_{h1,x}^{neq}\right)}^{2}+{\left(M_{h1,y}^{neq}\right)}^{2}}dxdy\\ S_{f3}=\int \sqrt{{\left(M_{f3,xxx}^{neq}\right)}^{2}+{\left(M_{f3,yyy}^{neq}\right)}^{2}+{\left(M_{f3,xyy}^{neq}\right)}^{2}+{\left(M_{f3,xxy}^{neq}\right)}^{2}}dxdy\\ S_{h2}=\int \sqrt{{\left(M_{h2,xx}^{neq}\right)}^{2}+{\left(M_{h2,yy}^{neq}\right)}^{2}+{\left(M_{h2,xy}^{neq}\right)}^{2}}dxdy \end{array}$$

They characterize the intensity of nonequilibrium effects at various physical levels in the fluid system at a certain moment. The results indicate that in the early stages of evolution, when the incident shock wave has just formed, the nonequilibrium effect intensity is significant. As the incident shock wave contacts the bottom wall and forms a reflected shock wave, there is a certain degree of enhancement in the nonequilibrium effects. Subsequently, as the reflected shock wave gradually takes shape, the nonequilibrium effect intensity remains within a certain range. Observing the overall trend, the nonequilibrium effect intensities $S_{h1}$ and $S_{h2}$ related to total energy show a clear decreasing trend from the formation of the incident shock wave to the generation of the reflected shock wave upon contacting the wall, while the change in the nonequilibrium effect intensity related to mass is relatively small. This is related to the fact that total energy contains richer information about motion. Thus, the evolution of nonequilibrium effect intensity can to some extent reflect the state of the flow system.

**Figure 14:**
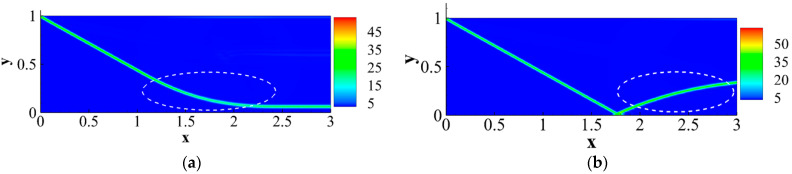
Magnitude of the density gradient of a regular reflection shock wave [44]: (**a**) $t=0.95t_{0}$ (**b**) $t=1.6t_{0}$.

During the unsteady solution process of shock wave reflection problems, it was observed that curved shock waves formed in both the incident shock wave and reflected shock wave regions, as shown in Figure 14. Consequently, an analysis of nonequilibrium effects was specifically conducted for curved shock waves. The focus was on understanding the similarities and differences in nonequilibrium effects between curved shock waves and straight shock waves. For detailed analysis, please refer to the literature [44].

**Figure 15:**
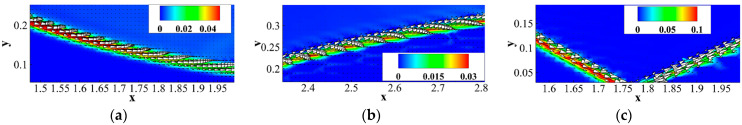
Vectors of nonequilibrium kinetic moments [44]: (**a**) $M_{f3,xxx}^{neq}$, $M_{f3,yyy}^{neq}$, t = 0.95t_0_ (**b**) $M_{f3,xxx}^{neq}$, $M_{f3,yyy}^{neq}$, t = 1.6t_0_ (**c**) $M_{f3,xxx}^{neq}$, $M_{f3,yyy}^{neq}$, t = 3.0t_0_.

**Figure 16:**
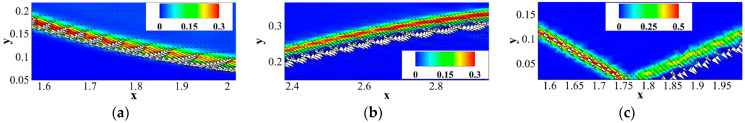
Vectors of nonequilibrium kinetic moments [44]: (**a**) $M_{h2,xx}^{neq}$, $M_{h2,yy}^{neq}$, t = 0.95t_0_ (**b**) $M_{h2,xx}^{neq}$, $M_{h2,yy}^{neq}$, t = 1.6t_0_ (**c**) $M_{h2,xx}^{neq}$, $M_{h2,yy}^{neq}$, t = 3.0t_0_.

To visually analyze the distribution characteristics of nonequilibrium effects more intuitively, the x and y components of the nonequilibrium moments can be considered as vector components for plotting. Figure 15 and Figure 16 show the distribution of some nonequilibrium effect vectors. The results indicate that nonequilibrium effects at different physical levels in the shock wave exhibit different distribution characteristics, showing a certain periodic trend along the shock wave. This periodic variation is particularly pronounced in the case of curved shock waves. The periodic variation at the straight shock wave is very small, which may be attributed to numerical oscillations near the shock wave. The significant periodic variation observed in curved shock waves is an “illusion” caused by the curved distribution. If viewed along the direction of the curved shock wave, nonequilibrium effects do not exhibit periodic distribution. Moreover, the distribution characteristics of nonequilibrium effects at the curved shock wave are related to the distribution characteristics of nonequilibrium effects at the two straight shock waves on either side. The main change is observed from the distribution characteristics of the straight shock wave on the incoming flow side to the distribution characteristics on the downstream side. Therefore, the observed significant changes in some nonequilibrium moments and the sustained conditions of others in the curved region in Figure 15 are related to the distribution characteristics of nonequilibrium effects at the straight shock waves on either side of the curved shock wave. For detailed analysis and discussion, please refer to the literature [44].

From the above research work, we can see that the characteristics and physical meaning of nonequilibrium effects of shock waves are studied through typical one- and two-dimensional shock wave problems. In practical engineering applications, there are often complex shock wave interference phenomena. It can be seen that the nonequilibrium effect is a very potential perspective for the study of shock wave phenomena. In the future, nonequilibrium effects can be used to study various typical shock wave interference phenomena, so as to obtain a deep understanding of the flow mechanism and develop methods to identify the characteristics of complex shock wave phenomena.

### 4.2. Shock Wave/Boundary Layer Interaction

#### 4.2.1. Oblique Shock Wave/Boundary Layer Interaction

Shock wave/boundary layer interaction (SWBLI) is the process where the bottom viscous boundary layer matches the external inviscid shock wave by thickening or separating, causing adverse pressure gradients. The SWBLI phenomenon has a significant impact on the flight safety and aerodynamic characteristics of aircraft. In the past two years, scholars have conducted mechanistic studies on the critical issue that must be considered in the design of hypersonic aircraft from a nonequilibrium perspective.

**Figure 17:**
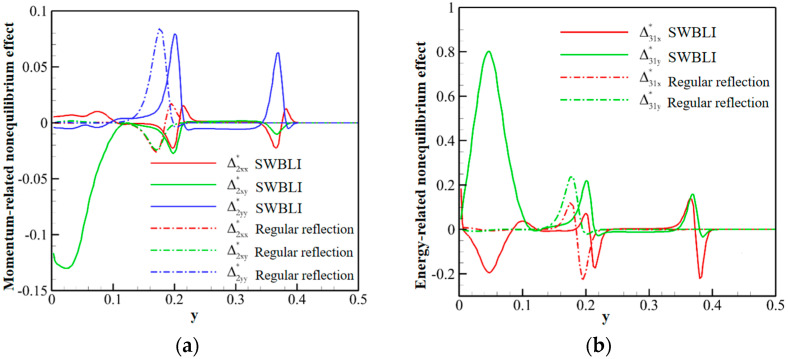
Distribution characteristics of nonequilibrium quantities [46]: (**a**) Distribution of mass-related nonequilibrium effects (**b**) Distribution of total energy-related nonequilibrium effects.

**Figure 18:**
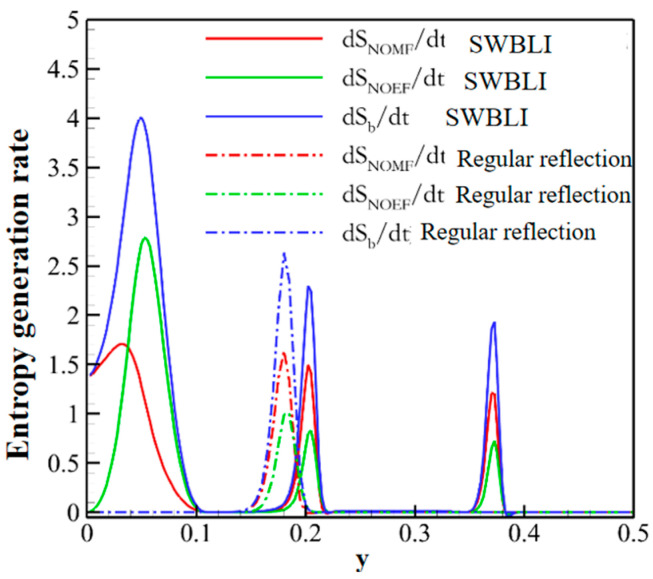
Distribution characteristics of entropy generation rates [46].

In the aspect of shock wave/laminar boundary layer interaction, Song et al. [46] primarily analyzed the entropy increase characteristics caused by nonequilibrium effects, such as viscosity and heat flux. The inflow Mach number simulated in their study was 5, and the gas was considered viscous and followed the Sutherland formula for viscosity. By observing the distribution of nonequilibrium effects at different positions along the vertical direction, as shown in Figure 17, and comparing it with two-dimensional regular reflected shock waves without considering viscosity, they found that the nonequilibrium effects in the former case are concentrated at the reflected shock wave, while in the latter case, they are concentrated at the reflected shock wave, reattachment shock wave, and the wall. Moreover, the total nonequilibrium intensity is highest near the wall. Examining the distribution of entropy generation rate in Figure 18 at these three positions, they observed three extreme states, indicating that shock waves and boundary layers contribute to the increased turbulence level in the flow field. At these critical positions, they conducted a detailed analysis of the contributions of total energy-related and mass-related nonequilibrium effects. They found that entropy increase at the shock wave surface is dominated by mass-related nonequilibrium effects, which are closely related to viscosity. On the other hand, near the wall, it is dominated by total energy-related nonequilibrium effects, which are inseparable from heat flux.

**Figure 19:**
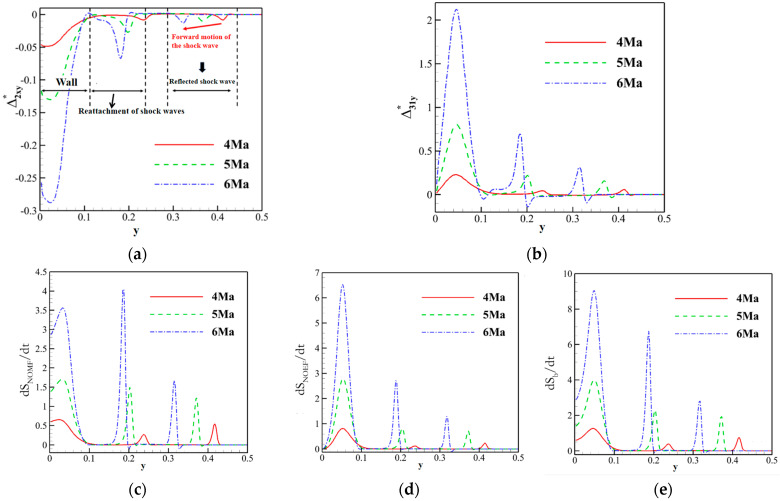
Distribution of nonequilibrium effects and entropy generation rates at different Mach numbers [46]: (**a**) Distribution of mass-related nonequilibrium effects (**b**) Distribution of total energy-related nonequilibrium effects (**c**) Distribution of entropy generation rate $dS_{NOMF}/dt$ (**d**) Distribution of entropy generation rate $dS_{NOEF}/dt$ (**e**) Distribution of entropy generation rate $dS_{b}/dt$.

Subsequently, they analyzed the distribution characteristics of the nonequilibrium effects shown in Figure 19 in the shock wave/laminar boundary layer interaction under different Mach numbers. They further investigated the influence of Mach number variations on thermodynamic nonequilibrium effects. The study revealed that at three critical positions, both total energy-related and mass-related nonequilibrium effects increase with an increase in the incoming Mach number. However, there are noticeable differences in the response of different positions to total energy-related nonequilibrium effects. Specifically, the vicinity of the reattachment shock wave is most affected by the viscous entropy generation rate, gradually overtaking the dominant position near the wall as the incoming Mach number increases. Whether at low or high Mach numbers, the strength relationships at different positions remain unchanged. Near the wall, entropy generation rates related to heat flux remain stronger than those near the reattachment shock wave and reflected shock wave even at high Mach numbers.

**Figure 20:**
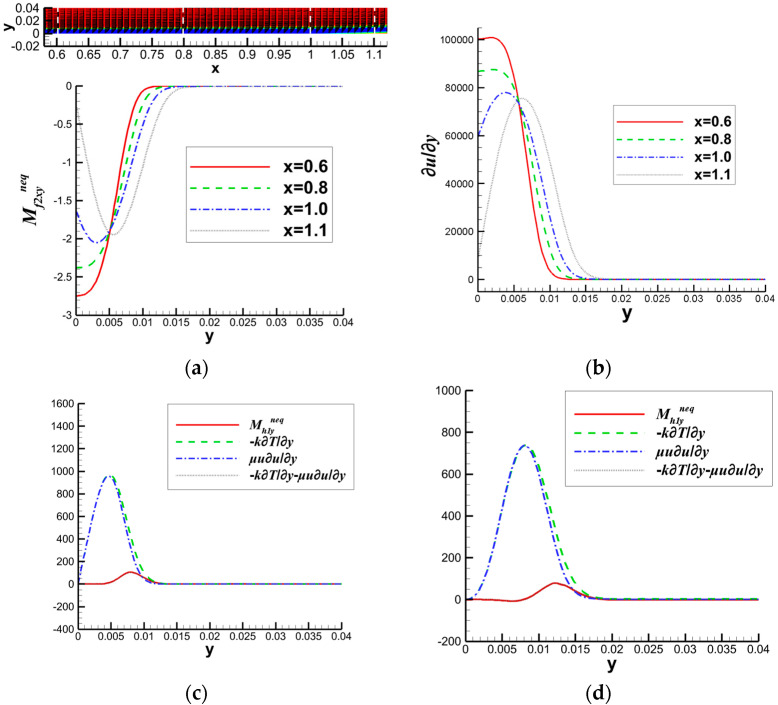
Distribution of nonequilibrium quantities and related macroscopic quantities [45]: (**a**) Distribution of mass-related nonequilibrium effects (**b**) Distribution of velocity gradient (**c**) Distributions of total-energy-related nonequilibrium effects and its elements at y = 0.6 (**d**) Distributions of total-energy-related nonequilibrium effects and its elements at y = 1.1.

Existing research on nonequilibrium effects in fluid dynamics is primarily based on solving LBM equations. However, LBM is not well-established for solving hypersonic flows involving shock waves and boundary layers. Research on hypersonic flow using LBM mainly focuses on exploring more optimal physical models. Faced with numerical stability issues in solving hypersonic flows with shock waves and boundary layers using LBM, BAO took a different approach and proposed a macroscopic-equation-based method for studying nonequilibrium effects related to SWBLI [45]. This method solves the macroscopic equations to analyze the flow field disturbed by shock waves and boundary layers, and then utilizes the obtained macroscopic quantities to derive corresponding nonequilibrium quantities. Taking a nonequilibrium perspective, they initially conducted a mechanistic exploration of the shock wave/laminar boundary layer interaction under Mach 2 inflow conditions. In the simulation, the left boundary serves as the inflow condition, specified as follows:${\left(T,u_{x},u_{y},p\right)}_{left}=\left(293,2.0\sqrt{\gamma RT,}0,657.5\right)$. By observing the characteristics of dominant nonequilibrium quantities related to mass and total energy, as illustrated in the Figure 20, and their connection with the non-uniformly distributed macroscopic quantities, they aimed to uncover the underlying reasons causing macroscopic phenomena.

**Figure 21:**
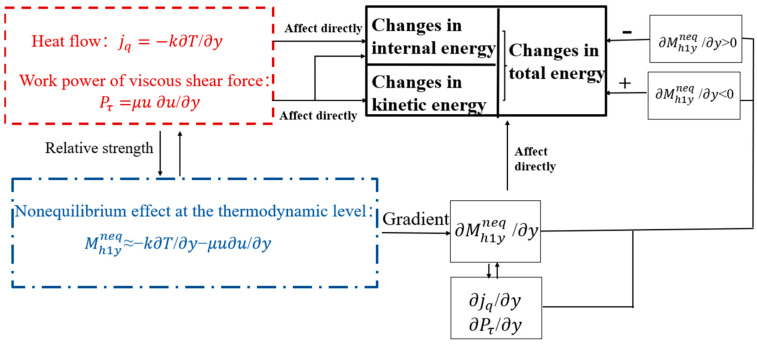
Boundary layer energy conversion relationship [45].

They further elucidated the energy conversion mechanism of boundary layer separation in SWBLI by analyzing the relationship between internal nonequilibrium effects in the boundary layer and energy conversion. The relationships are organized into the network shown in Figure 21.

**Figure 22:**
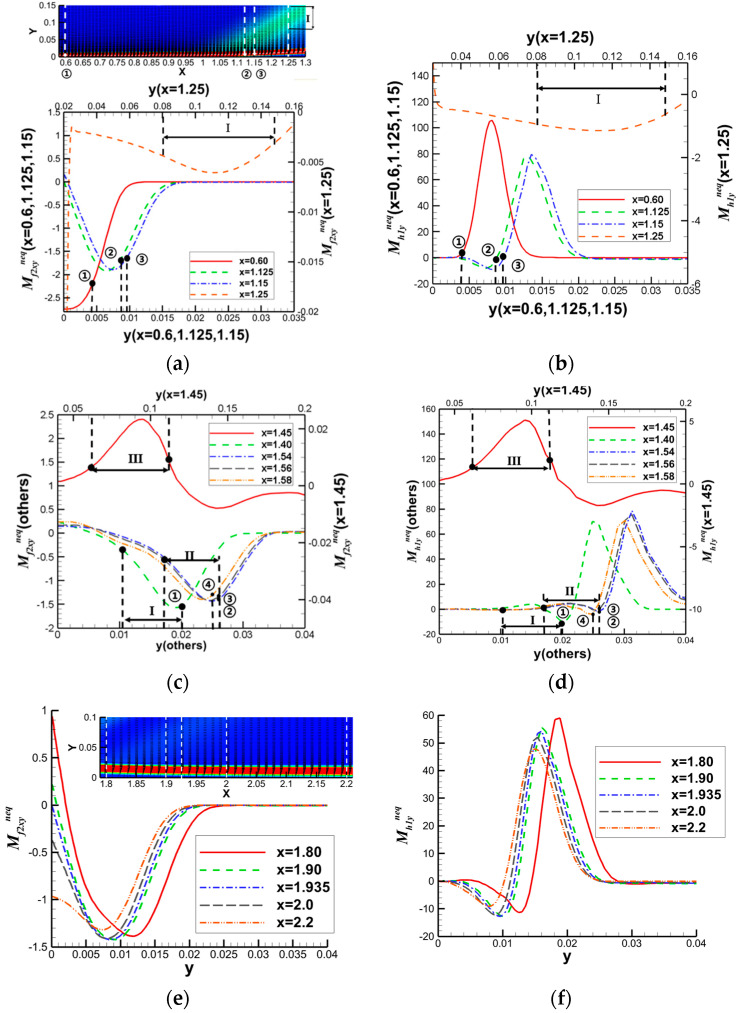
The distribution of nonequilibrium effects at different positions in the Mach 2 example [45]: (**a**) Distribution of mass-related nonequilibrium effects of separation shocks (**b**) Distributions of total-energy-related nonequilibrium effects of separation shocks (**c**) Distribution of mass-related nonequilibrium effects of incident shocks (**d**) Distributions of total-energy-related nonequilibrium effects of incident shocks (**e**) Distribution of mass-related nonequilibrium effects of reattachment shocks (**f**) Distributions of total-energy-related nonequilibrium effects of reattachment shocks.

Then, they further analyzed the flow characteristics of separation shocks, incident shocks, and reattachment regions based on the clue of the speed-of-sound lines that shock waves can reach within the boundary layer. The distribution of nonequilibrium quantities at these positions is illustrated in Figure 22, and specific analysis can be found in reference [45].

**Figure 23:**
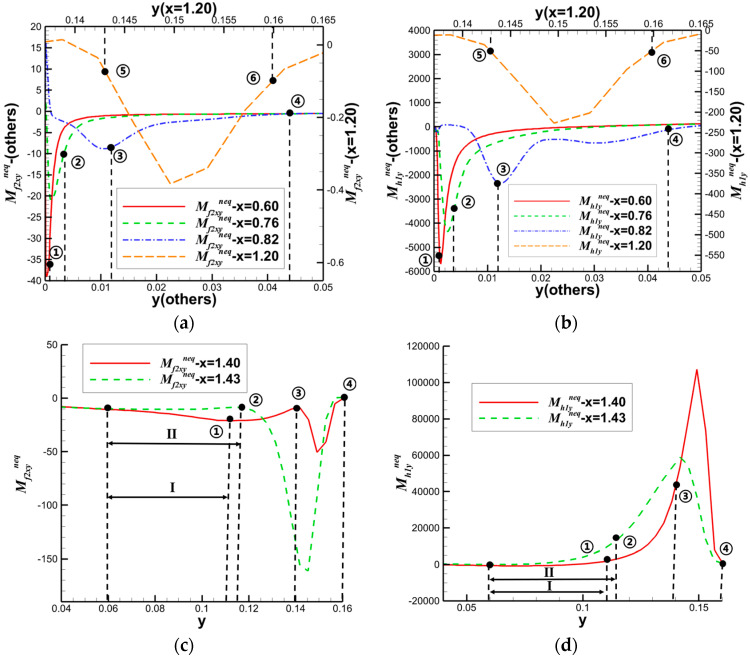
The distribution of nonequilibrium effects at different positions in the Mach 6 example [45]: (**a**) Distribution of mass-related nonequilibrium effects of separation shocks (**b**) Distributions of total-energy-related nonequilibrium effects of separation shocks (**c**) Distribution of mass-related nonequilibrium effects of incident shocks (**d**) Distributions of total-energy-related nonequilibrium effects of incident shocks.

To account for the transition from the laminar to turbulent boundary layer with an increasing freestream Mach number, they derived macroscopic expressions for different-level nonequilibrium effects when considering the influence of turbulence. They found that these expressions still corresponded to viscous stress terms and heat flux terms. However, the mass-related nonequilibrium effects include the impact of turbulent viscosity on viscous stress, and the total energy-related nonequilibrium effects, particularly the heat flux term, also encompass the influence of turbulence on energy conservation. In their analysis, they considered the interaction of a Mach 6 shock wave with a turbulent boundary layer, as depicted in Figure 23. In this particular case, the incoming flow condition at the left boundary is as follows: ${\left(T,u_{x},u_{y},p\right)}_{left}=\left(222.5,6.0\sqrt{\gamma RT,}0,2188\right)$.

They observed that, unlike the marginal impact of nonequilibrium effects on the boundary layer observed with a Mach 2 incident shock wave, the effects become more pronounced as the freestream Mach number increases. The shock wave intensifies further, and on the nonequilibrium effects, the incident shock wave notably influences the nonequilibrium effects in the boundary layers above and below the speed-of-sound line.

#### 4.2.2. Curved Shock Wave/Boundary Layer Interaction

**Figure 24:**
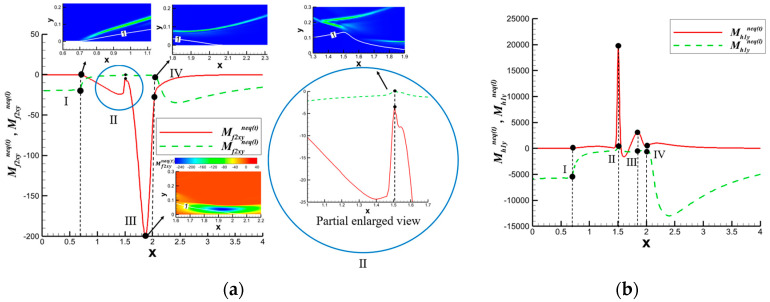
Distribution of nonequilibrium effects of Mach 6 CSWBLI on speed-of-sound lines: (**a**) Mass-related nonequilibrium effects (**b**) Total-energy-related nonequilibrium effects.

Building upon the aforementioned investigation into oblique shock/boundary layer interactions (OSWBLI), we extended our research to examine the effects of curved shock/boundary layer interactions (CSWBLI). These results, which are being presented for the first time, have not been published before. Given that the profiles of aircraft are often curved, curved shock waves induced are more prevalent compared to oblique shock waves. The fundamental distinction between OSWBLI and CSWBLI lies in the curvature variation of shock waves and the non-uniformity of the flow field behind the shock waves. The theoretical understanding of curved shock waves is continuously evolving, with Molder deriving the non-uniform first-order curved shock wave equations [50], and Shi et al. establishing connections between the parameters before and after the shock wave and the curvature of the shock wave [51,52]. Most existing studies predominantly explored CSWBLI from a macroscopic perspective. Recognizing the necessity of investigating CSWBLI, we adopted an analysis methodology similar to that of OSWBLI. We conducted further analysis on the positions where the nonequilibrium quantities, as shown in Figure 24, exhibited distinctive features in the interaction between curved shock waves and turbulent boundary layers. The partial dominant nonequilibrium moment components are represented by the symbols “$M_{f2,xy}^{\mathrm{neq}(l)}$$M_{h1,y}^{\mathrm{neq}(l)}$$M_{f2,xy}^{\mathrm{neq}(t)}$$M_{h1,y}^{\mathrm{neq}(t)}$”. The superscripts “l” and “t” denote the effects under laminar and turbulent conditions, respectively. By decomposing nonequilibrium effects into laminar and turbulent influence modes, we aim to provide readers with a clearer understanding of how these two modes contribute to nonequilibrium effects.

**Figure 25:**
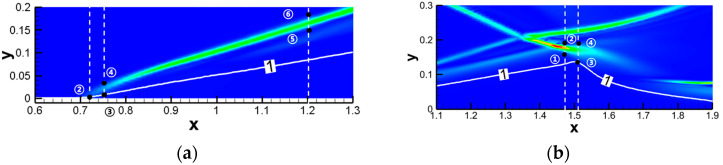
Schematic diagrams: (**a**) Separation shock (**b**) Transmitted shock.

**Figure 26:**
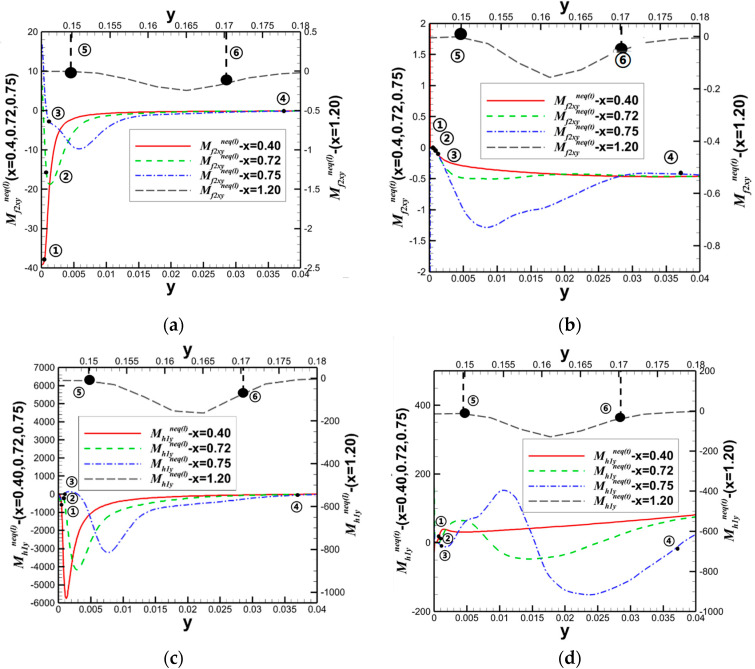
The distribution of nonequilibrium effects in laminar and turbulent modes of separation shock: (**a**) $M_{f2,xy}^{\mathrm{neq}(l)}$ (**b**) $M_{f2,xy}^{\mathrm{neq}(t)}$ (**c**) $M_{h1,y}^{\mathrm{neq}(l)}$ (**d**) $M_{h1,y}^{\mathrm{neq}(t)}$.

**Figure 27:**
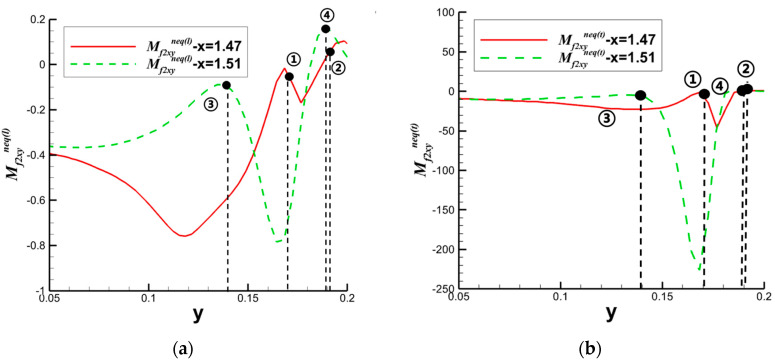

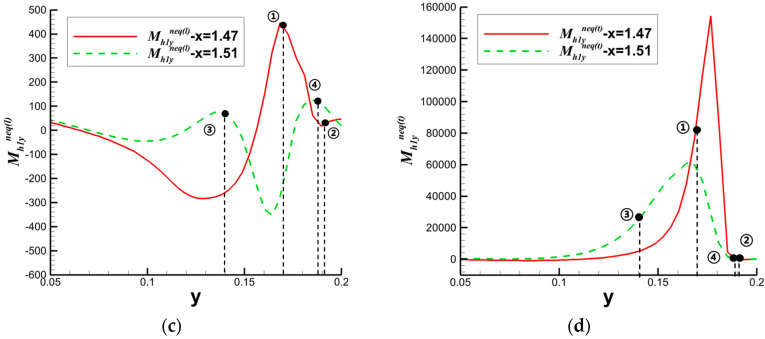
The distribution of nonequilibrium effects in laminar and turbulent modes of transmitted shock: (**a**) $M_{f2,xy}^{\mathrm{neq}(l)}$ (**b**) $M_{f2,xy}^{\mathrm{neq}(t)}$ (**c**) $M_{h1,y}^{\mathrm{neq}(l)}$ (**d**) $M_{h1,y}^{\mathrm{neq}(t)}$.

The schematic diagrams of the separation shock wave and transmitted shock wave are shown in Figure 25. The distribution of the dominant nonequilibrium moments at the positions of the separation shock wave and transmitted shock wave is illustrated in Figure 26 and Figure 27, respectively. The positions ➀–➅ shown in Figure 26 correspond to the position schematic of Figure 25a, and the positions ➀–➃ shown in Figure 27 correspond to the position schematic of Figure 25b. Through analysis, it was observed that, with the continuous action of shock waves on the boundary layer, the nonequilibrium effects related to turbulence are more than three orders of magnitude higher than those related to laminar flow. The turbulence-induced nonequilibrium effects gradually take over the dominant position.

**Figure 28:**
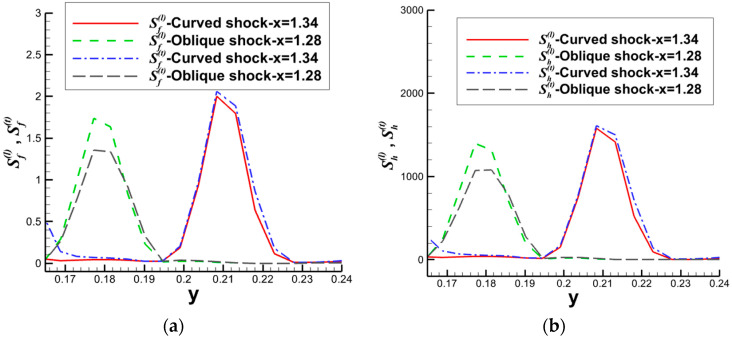
Comparison of nonequilibrium effect intensity between oblique shock wave and curved shock wave: (**a**) Mass-related nonequilibrium effect intensity (**b**) Total-energy-related nonequilibrium effect intensity.

After analyzing the connection between the distribution of nonequilibrium effects in CSWBLI and macroscopic phenomena, our focus shifted to understanding the characteristics of the separation region and its connection with nonequilibrium quantities caused by oblique shock waves and curved shock waves. To conduct such a test, it is necessary to ensure the consistency of the testing conditions between OSWBLI and CSWBLI, except for the differences in shock wave generator configurations. This includes consistent boundary layer development, as the boundary layer continuously thickens and laterally evolves, resulting in different separation characteristics of the boundary layer disturbed by shock waves. Comparing the adverse pressure gradient and nonequilibrium effect intensity at the three-wave points along the vertical direction, as shown in Figure 28, we found that, regardless of the adverse pressure gradient, total energy-related, or mass-related nonequilibrium effect intensity, the curved shock wave is greater than the oblique shock wave at the three-wave point positions. In other words, the intensity of nonequilibrium effects to some extent could characterize the shock wave intensity. This establishes a logical relationship where the stronger nonequilibrium effects induced by the curved shock wave lead to a stronger shock wave, resulting in a larger adverse pressure gradient and ultimately a higher macroscopic separation region.

**Figure 29:**
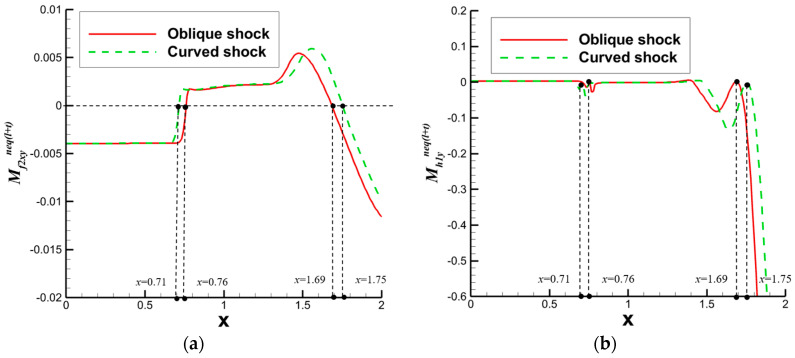
Comparison of the dominant nonequilibrium effects between CSWBLI and OSWBLI: (**a**) Mass-related nonequilibrium effect (**b**) Total-energy-related nonequilibrium effect.

Our team also observed that the enlargement of the boundary layer separation region in CSWBLI is manifested not only in the increased separation bubble height but also in the earlier onset of separation and a relatively delayed attachment point. By comparing the distribution of the dominant nonequilibrium quantities shown in Figure 29, it was found that before separation, the nonequilibrium effects are in a stable state. As the boundary layer exhibits a separated state under the influence of the shock wave, the nonequilibrium effects related to the viscous shear force tend to zero. The trend of approaching zero in CSWBLI is significantly earlier than in OSWBLI, and the span of nonequilibrium effects variation is also larger. This may be the reason for CSWBLI leading to a longer downstream separation distance.

The investigation of nonequilibrium effects on SWBLI has evolved from laminar to turbulent flows. Simultaneously, this study delves into CSWBLI. The research incrementally progresses, enhancing comprehension of this phenomenon from a nonequilibrium perspective. Due to the existing problems in the applicability of the supersonic LBM method for high Mach number flow, the existing literature is to extract nonequilibrium effects from the macroscopic equation. The current methods are not suitable for SWBLI of rarefied gases. It is necessary to establish the nonequilibrium effect analysis method by a unified method, such as the unified gas-kinetic scheme (UGKS) [53]. Additionally, findings from the existing literature suggest that nonequilibrium quantities related to shear stress can serve as criteria for boundary layer separation, potentially playing a pivotal role in shock/boundary layer control. These endeavors aim to deepen our understanding of shock-boundary layer interactions, providing robust support for the advancement of related fields. Moreover, the nonequilibrium kinetic approach is expected to be further applied to the aerodynamic design and optimization of aircraft and engines.

### 4.3. Detonation Wave

Detonation is a chemical reaction propagation process accompanied by a large release of energy, where the leading edge of the reaction zone forms a shock wave with supersonic motion, known as a detonation wave. Pulse detonation engines [54,55], rotating detonation engines [56,57], oblique detonation engines [58,59], or curved detonation engines [60,61] utilize this phenomenon to generate thrust using high-temperature and high-pressure combustion gases. They exhibit characteristics such as a simple structure and a high thrust-to-weight ratio relative to traditional engines, making them a highly promising class of propulsion systems for aerospace applications. Scholars have conducted a series of research studies on the mechanism of detonation waves from the perspective of nonequilibrium behavior.

**Figure 30:**
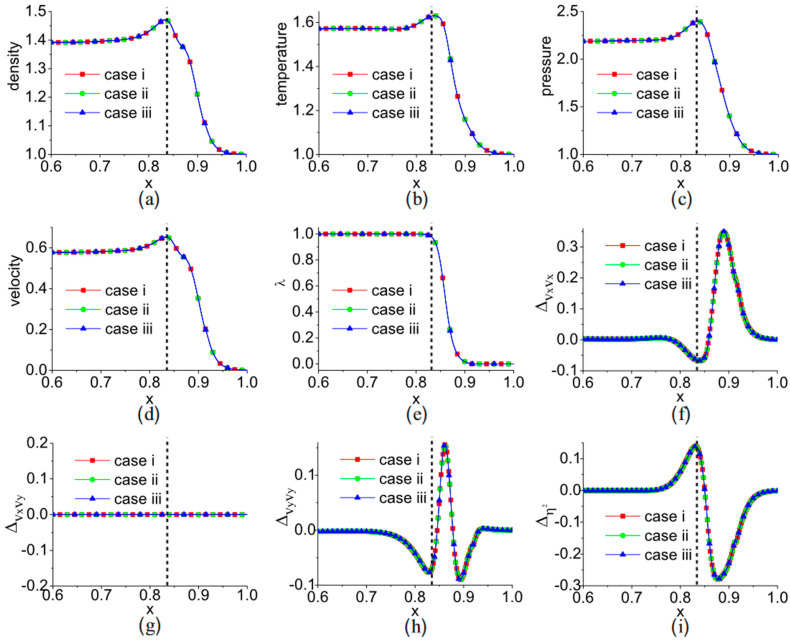

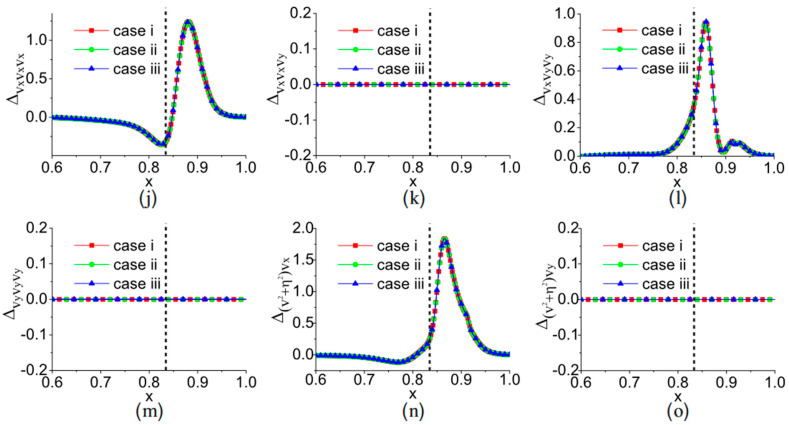
Macroscopic physical quantities and nonequilibrium quantities in an unsteady detonation [3]: (**a**) ρ (**b**) T (**c**) p (**d**) $u_{x}$ (**e**) λ (**f**) $\Delta_{v_{x}v_{x}}$ (**g**) $\Delta_{v_{x}v_{y}}$ (**h**) $\Delta_{v_{y}v_{y}}$ (**i**) $\Delta_{\eta^{2}}$ (**j**) $\Delta_{v_{x}v_{x}v_{x}}$ (**k**) $\Delta_{v_{x}v_{x}v_{y}}$ (**l**) $\Delta_{v_{x}v_{y}v_{y}}$ (**m**) $\Delta_{v_{y}v_{y}v_{y}}$ (**n**) $\Delta_{(v^{2}+\eta^{2})v_{x}}$ (**o**) $\Delta_{(v^{2}+\eta^{2})v_{y}}$.

In 2015, Xu et al. investigated the nonequilibrium behavior near one-dimensional detonation waves [3]. Figure 30 illustrates the macroscopic and nonequilibrium quantities near the detonation wave. The initially simulated flow field for the one-dimensional detonation simulation is as follows: ${\left(\rho ,T,u_{x},\gamma \right)}_{left}=\left(1.38837,1.57856,0.57735,1\right)$, ${\left(\rho ,T,u_{x},\gamma \right)}_{right}=\left(1,1,0,0\right)$. The results show that the sum of the deviations from the equilibrium of internal energy in the x-direction, internal energy in the y-direction, and additional degree of freedom energy is zero. This is because, when the system is not in thermal equilibrium, the energies associated with different degrees of freedom may deviate in different directions due to molecular collisions. In this one-dimensional flow in the x-direction, shear effects and energy flux associated with the y-direction are in equilibrium. They also observed that while the system’s viscosity or thermal conductivity may reduce local thermal nonequilibrium, it increases the overall thermal nonequilibrium near the detonation wave.

**Figure 31:**
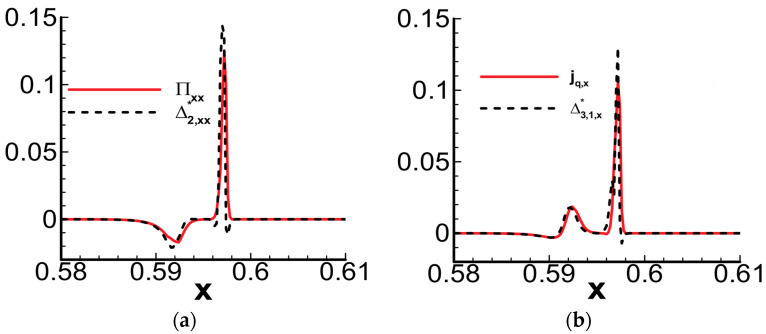
Comparisons of viscous stress, heat flux, and nonequilibrium quantities [29]: (**a**) Viscous stress and nonequilibrium quantity (**b**) Heat flux and nonequilibrium quantity.

Figure 31 presents the quantitative comparison results of viscous stress, heat flux, and their corresponding nonequilibrium quantities by Zhang et al. [29]. The simulated initial conditions are consistent with the one-dimensional detonation case mentioned earlier in this paper. From the figure, it can be observed that viscous stress and heat flux are numerically consistent with their respective nonequilibrium quantities. Additionally, these nonequilibrium quantities are directly derived from the Boltzmann equation, providing a more accurate description of flow phenomena with strong nonequilibrium effects, such as detonation waves.

**Figure 32:**
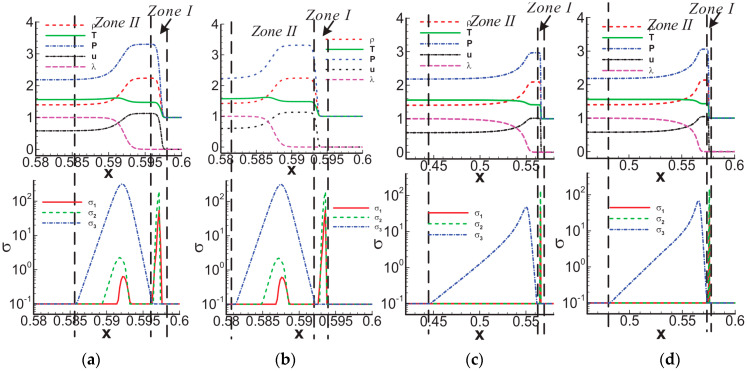
Three kinds of profiles of entropy productions [29]: (**a**–**d**) Entropy production in the process of detonation in different case.

They further derived an expression for entropy increase based on the mathematical relationships among viscous stress, heat flux, and their corresponding nonequilibrium quantities. From this expression, it becomes evident that entropy increase is attributed to both thermodynamic nonequilibrium effects and chemical reactions. The computational results for detonation waves under various conditions (refer to Figure 32) reveal that entropy increase is primarily governed by chemical reactions, with the contribution from thermodynamic nonequilibrium effects being relatively small. Moreover, among the latter two factors, the entropy increase induced by mass-related nonequilibrium effects is significantly larger than that from total energy-related nonequilibrium effects.

**Figure 33:**
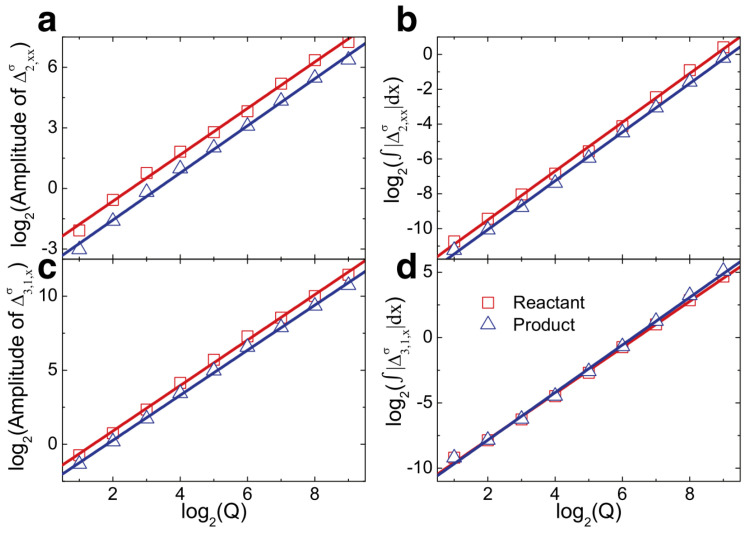
Nonequilibrium effects versus the chemical heat release [30]: (**a**) Amplitude of $\Delta_{2,xx}^{\sigma }$ (**b**) $\int \left|\Delta_{2,xx}^{\sigma }\right|dx$ (**c**) Amplitude of $\Delta_{3,1,x}^{\sigma }$ (**d**) $\int \left|\Delta_{3,1,x}^{\sigma }\right|dx$.

Lin et al. investigated the impact of chemical heat and relaxation time on the detonation wave mechanism using nonequilibrium analysis [30]. The initially simulated flow field in this study was: ${\left(\rho ,u_{x},p\right)}_{left}=\left(1.48043,0.81650,3.05433\right)$, ${\left(\rho ,u_{x},p\right)}_{right}=\left(1,0,1\right)$. Figure 33 illustrates the relationship between nonequilibrium effects and chemical heat. Both the local and global thermodynamic nonequilibrium effects of detonation waves intensify with an increase in chemical heat. Upon the logarithmic transformation of chemical heat and nonequilibrium effects, an approximately linear relationship between the two is observed. Remarkably, this characteristic holds for both reactants and products of the chemical reactions.

**Figure 34:**
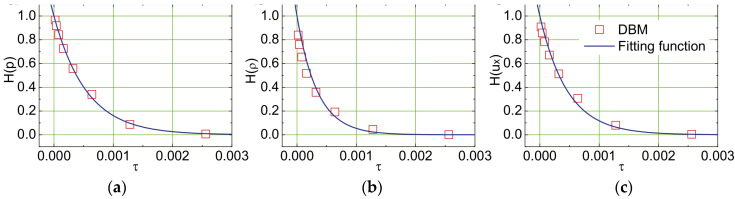
The peak heights of pressure, density, and horizontal velocity versus the relaxation time [30]: (**a**) Pressure (**b**) Density (**c**) Horizontal velocity.

**Figure 35:**
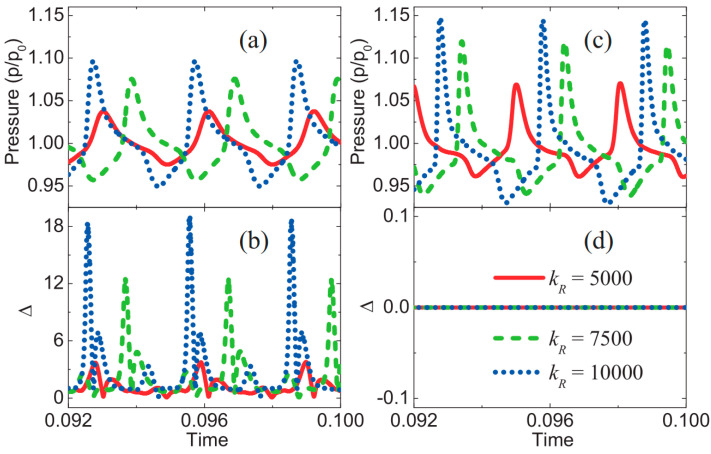
(**a**) Shock pressure (**b**) Nonequilibrium strength with nonequilibrium effects (**c**) Shock pressure (**d**) Nonequilibrium strength without nonequilibrium effects [31].

Additionally, regarding the influence of relaxation time on controlling the transition of fluid systems toward equilibrium velocities, Lin et al. investigated the peak heights of macroscopic quantities such as the detonation wave density and temperature, along with nonequilibrium effects under different relaxation times. They discovered that as the relaxation time increases, the global nonequilibrium effects of the fluid system increase, resulting in lower peak heights and broader widths of the detonation wave. Figure 34 illustrates the relationship between the peak heights of macroscopic quantities in detonation waves and relaxation time. Subsequently, they compared the macroscopic quantities and nonequilibrium effects of detonation waves, considering and neglecting nonequilibrium effects, respectively, as depicted in Figure 35. The results still conform to this pattern [31].

**Figure 36:**
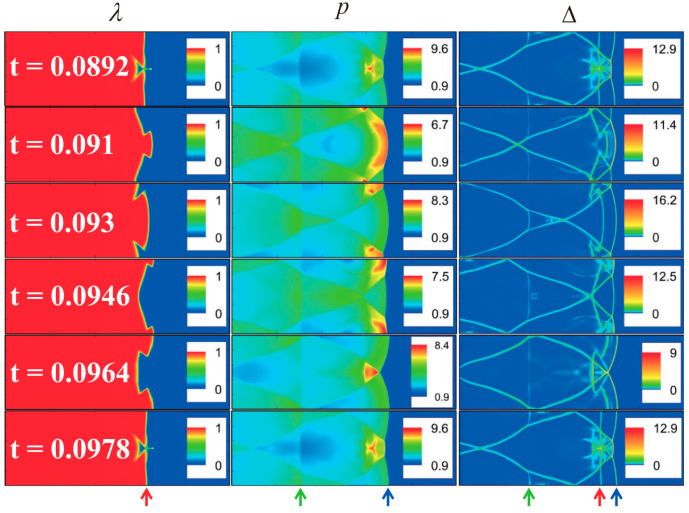
Snapshots of the reaction process (**left**), pressure (**middle**), and nonequilibrium effect (**right**) during a period of unsteady detonation from top to bottom, respectively [31].

Figure 36 presents the results of the unsteady detonation wave within one cycle, showing the reaction process, pressure, and nonequilibrium effects [31]. During the evolution, the reaction layer exhibits a fluctuating concave-convex shape, and the peak pressure repeatedly varies in the y-direction center and both sides. Examining the distribution of nonequilibrium effects reveals pronounced nonequilibrium states in regions of high reaction and pressure gradients, specifically highlighting strong nonequilibrium effects at the leading edge of the detonation wave and the reaction zone. Additionally, transverse waves exhibit noticeable nonequilibrium effects.

**Figure 37:**
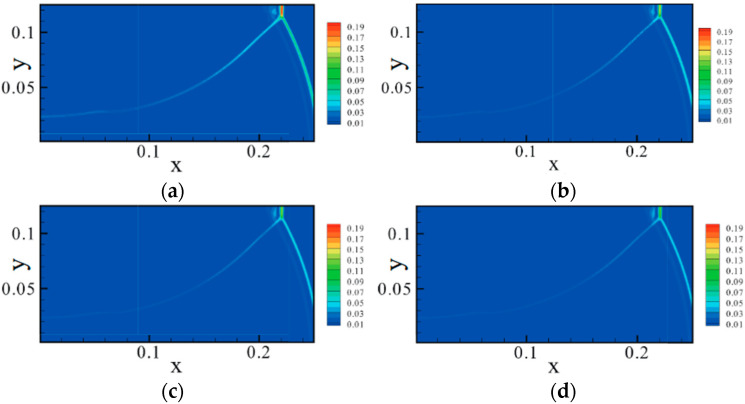
Distribution of nonequilibrium effects with different Pr numbers [47]: (**a**) Pr = 0.15 (**b**) Pr = 0.5 (**c**) Pr = 1.0 (**d**) Pr = 1.5.

In the evolution of two-dimensional detonation waves, Shan et al. [47] investigated the impact of the Prandtl number (Pr) on nonequilibrium effects in detonation waves, as shown in Figure 37. The simulated initial flow field in this study is as follows: ${\left(\rho ,u_{x},u_{y},T,\lambda \right)}_{left}=\left(1.388,0.577,0,1.579,1\right)$ (upper left and lower left plot A and B of the computational domain), ${\left(\rho ,u_{x},u_{y},T,\lambda \right)}_{right}=\left(1,0,0,1,0\right)$ (others). They observed that variations in the Pr have minimal influence on the propagation speed of detonation waves. However, with a decrease in the Pr, total energy-related nonequilibrium effects increase.

**Figure 38:**
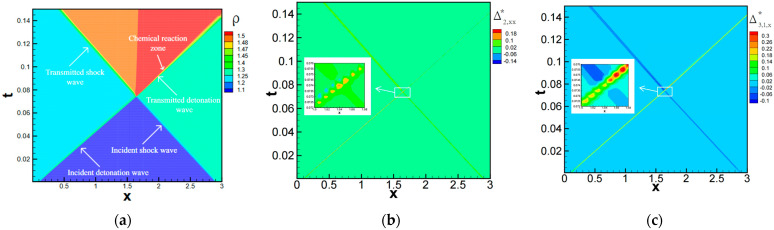
Distribution of [47]: (**a**) Density (**b**) Mass-related nonequilibrium effect (**c**) Total-energy-related nonequilibrium effect.

Moreover, the mutual interference between shock waves and detonation waves is prevalent in the internal flow of aircraft. Shan [47] conducted a study on the nonequilibrium characteristics of this phenomenon. Figure 38 illustrates the density contour and distribution of nonequilibrium effects after their interaction. In this scenario, there is a sudden change in the nonequilibrium moments ahead of the shock and detonation waves, and their collision results in varying degrees of increase in nonequilibrium quantities at different physical levels.

In the case of detonation waves, the nonequilibrium effect also brings a new mesoscopic perspective to the macroscopic approach. From the point of view of the nonequilibrium effect, the discussion of the detonation wave is still carried out through the nonequilibrium effect related to viscosity and heat flow. Combustion is one of the core phenomena of the detonation wave. The use of mesoscopic dynamics to extract the nonequilibrium effects related to the chemical reaction of the detonation wave will provide a deeper understanding of the detonation wave. Moreover, the present studies are only for simple cases, which are quite different from the actual engine combustion chamber. The actual working conditions put forward have higher requirements for the solving ability of the mesoscopic kinetics method.

## 5. Summary and Discussion

This article is a review of a research method that describes the nonequilibrium effects of shock waves from the perspective of molecular motion. The method is based on the Boltzmann-BGK equation or MRT-Boltzmann equation, which reflects the deviation of the fluid system from local equilibrium through nonequilibrium moments. A detailed analysis and discussion of nonequilibrium moments are provided, including their role in the fluid system, the relationship between nonequilibrium and equilibrium moments based on Chapman-Enskog analysis, and the physical meaning of nonequilibrium effects based on kinetic theory. Subsequently, the article demonstrates the application of this method to study the nonequilibrium behavior of phenomena such as shock waves, SWBLI, and detonation waves. The following provides some explanations for this method:

1. The method presented in the article offers additional flow field information beyond macroscopic variables from the perspective of molecular nonequilibrium motion for shock wave systems, enhancing the understanding of shock wave phenomena.
2. The discussion in this article uses the Navier-Stokes level as an example, but the method is not limited to the Navier-Stokes level. It is applicable to LBM models with higher physical accuracy.
3. Nonequilibrium effects are the root cause of the deviation of fluid systems from equilibrium. In the absence of chemical reactions, they are macroscopically related to the viscous stress and heat flux in fluid flow. In other words, viscous stress and heat flux act as the “instigators” of nonequilibrium behavior in fluid systems.
4. Nonequilibrium effects represent a kind of “instability” of molecular motion relative to local equilibrium, and their numerical magnitude reflects the strength of the system’s “instability”.
5. Strong nonequilibrium effects exist within shock waves. Interactions such as shock waves with solid walls, contact discontinuities, expansion waves, and boundary layers lead to variations in nonequilibrium behavior.

We suggest that the nonequilibrium effect can be further studied in the following aspects:

1. Delve deeper into exploring the nonequilibrium effects of fluid systems. Utilize information on nonequilibrium behavior to reveal the formation mechanisms of flow phenomena at the mesoscopic level, further enabling the prediction of complex fluid flow systems.
2. Trace the origin of fluid systems deviating from equilibrium using nonequilibrium moments. Employ nonequilibrium effects to control fluid flow systems and integrate them into the aerodynamic design of high-speed aircraft and engines.
3. Extend the application of this research method to complex flow phenomena such as turbulence, aerodynamic heating, rarefied flow, etc.
4. For problems with source terms, such as chemical reaction, further develop a nonequilibrium effect extraction approach to obtain the source-term related to nonequilibrium effects.
5. Develop an approach for the nonequilibrium effect based on the unified mesoscopic kinetic method applicable to simulating fluids from continuous to rarefied.

## Figures and Tables

**Table 1:** Physical meaning of equilibrium kinetic moments of mass density.

Equilibrium Moments	Physical Meaning	Dimension (Unit)
*M*_0_ (*f^eq^*)	Molecular mass density	kg m^−3^
*M*_1,*i*_ (*f^eq^*)	Molecular momentum density in the *i*-direction	N s m^−3^
*M*_2,*ij*_ (*f^eq^*), *i* = *j*	Molecular translational kinetic energy density in the *i*-direction at equilibrium	N m^−2^ or J m^−3^
*M*_2,*ij*_ (*f^eq^*), *i* ≠ *j*	Moments of the first-order motion state of $f_{\alpha }^{eq}e_{\alpha i}$ in the *j*-direction	N m^−2^ or J m^−3^
*M*_3,*ijk*_ (*f^eq^*), *i* = *j* = *k*	Moments of the first-order motion state of *f_α_^eq^e_αi_e_αi_*	N m^−1^ s^−1^ or J m^−2^ s^−1^
*M*_3,*ijk*_ (*f^eq^*), *i* = *j* ≠ *k*	Moments of the first-order motion state of *f_α_^eq^e_αi_e_αi_* in the *k*-direction	N m^−1^ s^−1^ or J m^−2^ s^−1^
*M*_3,*ijk*_ (*f^eq^*), *i* ≠ *j* ≠ *k*	The moment of the first-order motion state in the *j*-direction and the first-order motion state in the *k*-direction for *f_α_^eq^e_αi_*	N m^−1^ s^−1^ or J m^−2^ s^−1^

**Table 2:** Physical meaning of equilibrium kinetic moments of total energy density.

Equilibrium Moments	Physical Meaning	Dimension (Unit)
*M*_0_ (*h^eq^*)	Total energy density of molecules	J m^−3^ or N m^−2^
*M*_1,*i*_ (*h^eq^*)	The moment of the first-order motion state in the *i*-direction for *h_α_^eq^*	J m^−2^ s^−1^ or N m^−1^ s^−1^
*M*_2,*ij*_ (*h^eq^*), *i* = *j*	The moment of the second-order motion state in the *i*-direction for *h_α_^eq^*	J m^−1^ s^−2^ or N s^−2^
*M*_2,*ij*_ (*h^eq^*), *i* ≠ *j*	The moment of the first-order motion state in the *i*-direction and the first-order motion state in the *j*-direction for *h_α_^eq^*	J m^−1^ s^−2^ or N s^−2^

## Data Availability

Data are contained within the article.
